# Independent Evolution of the MYB Family in Brown Algae

**DOI:** 10.3389/fgene.2021.811993

**Published:** 2022-02-04

**Authors:** Qiangcheng Zeng, Hanyu Liu, Xiaonan Chu, Yonggang Niu, Caili Wang, Gabriel V. Markov, Linhong Teng

**Affiliations:** ^1^ College of Life Sciences, Dezhou University, Dezhou, China; ^2^ Sorbonne Université, CNRS, Integrative Biology of Marine Models (LBI2M), Station Biologique de Roscoff (SBR), Roscoff, France

**Keywords:** MYB, gene family, transcription factor, evolution, brown algae

## Abstract

Myeloblastosis (MYB) proteins represent one of the largest families of eukaryotic transcription factors and regulate important processes in growth and development. Studies on MYBs have mainly focused on animals and plants; however, comprehensive analysis across other supergroups such as SAR (stramenopiles, alveolates, and rhizarians) is lacking. This study characterized the structure, evolution, and expression of MYBs in four brown algae, which comprise the biggest multicellular lineage of SAR. Subfamily 1R-MYB comprised heterogeneous proteins, with fewer conserved motifs found outside the MYB domain. Unlike the SHAQKY subgroup of plant 1R-MYB, THAQKY comprised the largest subgroup of brown algal 1R-MYBs. Unlike the expansion of 2R-MYBs in plants, brown algae harbored more 3R-MYBs than 2R-MYBs. At least ten 2R-MYBs, fifteen 3R-MYBs, and one 6R-MYB orthologs existed in the common ancestor of brown algae. Phylogenetic analysis showed that brown algal MYBs had ancient origins and a diverged evolution. They showed strong affinity with stramenopile species, while not with red algae, green algae, or animals, suggesting that brown algal MYBs did not come from the secondary endosymbiosis of red and green plastids. Sequence comparison among all repeats of the three types of MYB subfamilies revealed that the repeat of 1R-MYBs showed higher sequence identity with the R3 of 2R-MYBs and 3R-MYBs, which supports the idea that 1R-MYB was derived from loss of the first and second repeats of the ancestor MYB. Compared with other species of SAR, brown algal MYB proteins exhibited a higher proportion of intrinsic disordered regions, which might contribute to multicellular evolution. Expression analysis showed that many MYB genes are responsive to different stress conditions and developmental stages. The evolution and expression analyses provided a comprehensive analysis of the phylogeny and functions of MYBs in brown algae.

## Introduction

The MYB (myeloblastosis) gene family is one of the largest families of transcription factors (TFs) found in nearly all eukaryotic organisms. MYBs play important roles in a variety of critical processes, such as regulating organism development, metabolism, cell morphology, and response to various stresses (Dubos et al., 2010; Cao et al., 2020). MYB proteins are characterized by a highly conserved DNA-binding domain (DBD), which comprises one or several adjacent repeats (R) of about 50–53 amino acids and forms three α helixes (Lipsick 1996). Each R contains three regularly spaced conserved tryptophans (W) and eight other residues to form the hydrophobic pocket that interacts with targeting DNA. The second and third helices fold into helix-turn-helix structures to bind the major groove of DNA (Ogata et al., 1992; Ogata et al., 1994; Kranz et al., 2000).

MYB TFs are classified based on the number of repeats. 1R-MYBs or MYB-related proteins contain the R sequence only once and comprise a heterogeneous subfamily (Feldbrügge et al., 1997). It was hypothesized that 1R-MYB diversified through expansions and domain shuffling (Du et al., 2013). The 1R domain was conserved and under strong purifying selection. Many 1R-MYBs were identified to function in diverse biological processes, such as transcriptional regulation, circadian clock-associated regulation, telomeric repeat-binding, and stress responses (Du et al., 2013). 2R-MYBs form the most common and expanded group in angiosperms, with more than 170 members reported in *Arabidopsis thaliana* (Stracke et al., 2001), 116 in *Capsicum* (Arce-Rodríguez et al., 2021), and 222 in *Musa acuminata* (Tan et al., 2020), while fewer than ten 3R-MYBs were found per species. Generally, seed plants have more R2R3 copies than ferns, and ferns have more copies than lycophytes (Hernández-Hernández et al., 2021). A vast number of plant 2R-MYBs have been shown to play important roles in plant-specific metabolic, morphogenic, or stress pathways (Ito 2005; Du et al., 2013). Through comparison with *Arabidopsis* genes, R2R3 MYB genes from four species of Solanaceae were found to be lost or gained during the divergence of the Rosid and Asterid lineages (Gates et al., 2016). Asymmetric gene duplication events in the 10 subfamilies of R2R3-MYBs contributed to the expansion of 2R-MYBs in embryophytes. Six duplication events produced seven clades of the largest subfamily VIII, while other subfamilies of 2R-MYB were less expanded (Jiang and Rao 2020).

3R-MYBs involved in cellular proliferation and differentiation are common in animals (Ramsay and Gonda 2008) and were also reported in the cellular slime mold (Otsuka and Van Haastert 1998). Three 3R-MYBs were found in most vertebrates, while most invertebrate genomes encode a single one (Lipsick et al., 2001; Davidson et al., 2012; Campanini et al., 2015). The three vertebrate MYB genes *c-Myb*, *A-MYB*, and *B-MYB* arose by two rounds of regional genomic duplications (Davidson et al., 2012). Aberrant activation of vertebrate MYBs could cause human malignancies (Cicirò and Sala 2021). Plants only make a limited number of 3R proteins compared to hundreds of 2R ones. Three subgroups of 3R-MYB proteins arose through two segmental duplication events before the common ancestor of angiosperms (Feng et al., 2017). Plant 3R-MYBs recognize mitosis-specific activator and function in both cell cycle regulation and abiotic stress responses (Ma et al., 2009). It was once assumed that plant 3R genes are distantly related to animal 3R genes and that plant 3R genes produce diverse and numerous R2R3 genes in plants by loss of R1 (Braun and Grotewold 1999; Dias et al., 2003). However, the DBD sequences of 3R-MYB from *Physcomitrella patens* and *Arabidopsis* showed high similarity to those of animal 3R-MYBs and less similarity to 2R-MYBs from plants, suggesting that 3R-MYBs existed in the common ancestor of animals and plants (Kranz et al., 2000). A single copy gene encoding a member of the 4R-MYB class was also present in many plants (Stracke et al., 2001; Yanhui et al., 2006). One 4R-MYB identified in *Arabidopsis* was a small nuclear RNA (snRNA)-activating protein complex subunit and initiated the transcription of snRNAs. Like 3R-MYB, this type of MYB showed evolutionary conservation in a broad range of eukaryotes (Thiedig et al., 2021).

Up to now, genome-wide analyses of 1R-MYB, 2R-MYB, and 3R-MYB proteins have been conducted in numerous eukaryotic species. However, comprehensive analysis of the MYB proteins in brown algae and other SAR (stramenopiles, alveolates, and rhizarians) lineages is still lacking. Brown algae comprise the biggest multicellular lineage in the SAR supergroup, which originated from secondary endosymbiotic events, in which red and green algae were engulfed by a eukaryotic heterotroph (Moustafa et al., 2009). The gene transfer from the endosymbiont to the host built a complex genomic mosaic in the SAR clade (Dorrell et al., 2017; Horák et al., 2020). The origin of brown algal MYBs and their evolutionary relationships with MYBs of other phyla remain unknown. Did they come from plastids of red or green algae? Or from the heterotrophic host? What about the relationships among the different kinds of MYB subfamilies? Brown macroalgae are the dominant primary producers in many benthic marine habitats with high ecological and economic significance (Teng et al., 2017a; Xu et al., 2017). The completion of genome sequencing projects for the model brown algae *Ectocarpus siliculosus* (Cock et al., 2010), *Saccharina japonica* (Ye et al., 2015), *Cladosiphon okamuranus*, and *Nemacystus decipiens* (Nishitsuji et al., 2016; Nishitsuji et al., 2019) facilitates exhaustive inventories of the MYB genes and cross-phyla comparisons. In the present study, MYB genes were identified in those brown algae, and their evolutionary relationships were explored. The analysis will provide a detailed picture of the MYB gene family and address the above questions, as well as provide a reference for further functional investigation of these genes in the SAR lineage.

## Results

### Global Identification of MYB Proteins From Brown Algae and Other Species

Overall, 172 brown algal MYB genes were identified, roughly equally distributed among the four investigated species: *E. siliculosus* (45), *S. japonica* (37), *C. okamuranus* (44), and *N. decipiens* (46). According to their numbers of repeat units, the 172 MYB genes were classified into four types: 1R-MYBs (62), 2R-MYBs (38), 3R-MYBs (58), and more R-MYBs (4R-MYB, 5R-MYB, and 6R-MYB). 1R-MYB, 2R-MYB, 3R-MYB, and 6R-MYB exist in all four species. The lengths of the proteins encoded by these 172 MYB genes ranged from 79 (Ec-12_000700 in *E. siliculosus*) to 1,967 amino acids (g14532.t1 in *N. decipiens*), with an average of 787 amino acids. The p*I* also varied greatly from 4.13 (SJ16925 in *S. japonica*) to 11.82 (SJ21668 in *S. japonica*), indicating their potential functional diversity. The molecular weight ranged from 8.738 kDa (Ec-12_000700 in *E. siliculosus*) to 205.14 kDa (g14532.t1 in *N. decipiens*), with an average of 82 kDa. In addition, prediction of subcellular addressing signal sequences showed that most genes localized in the nucleus and had no transmembrane helix. Detailed information is listed in Supplementary Table S1.

### Characteristics of the MYB Domain

**FIGURE 1:**
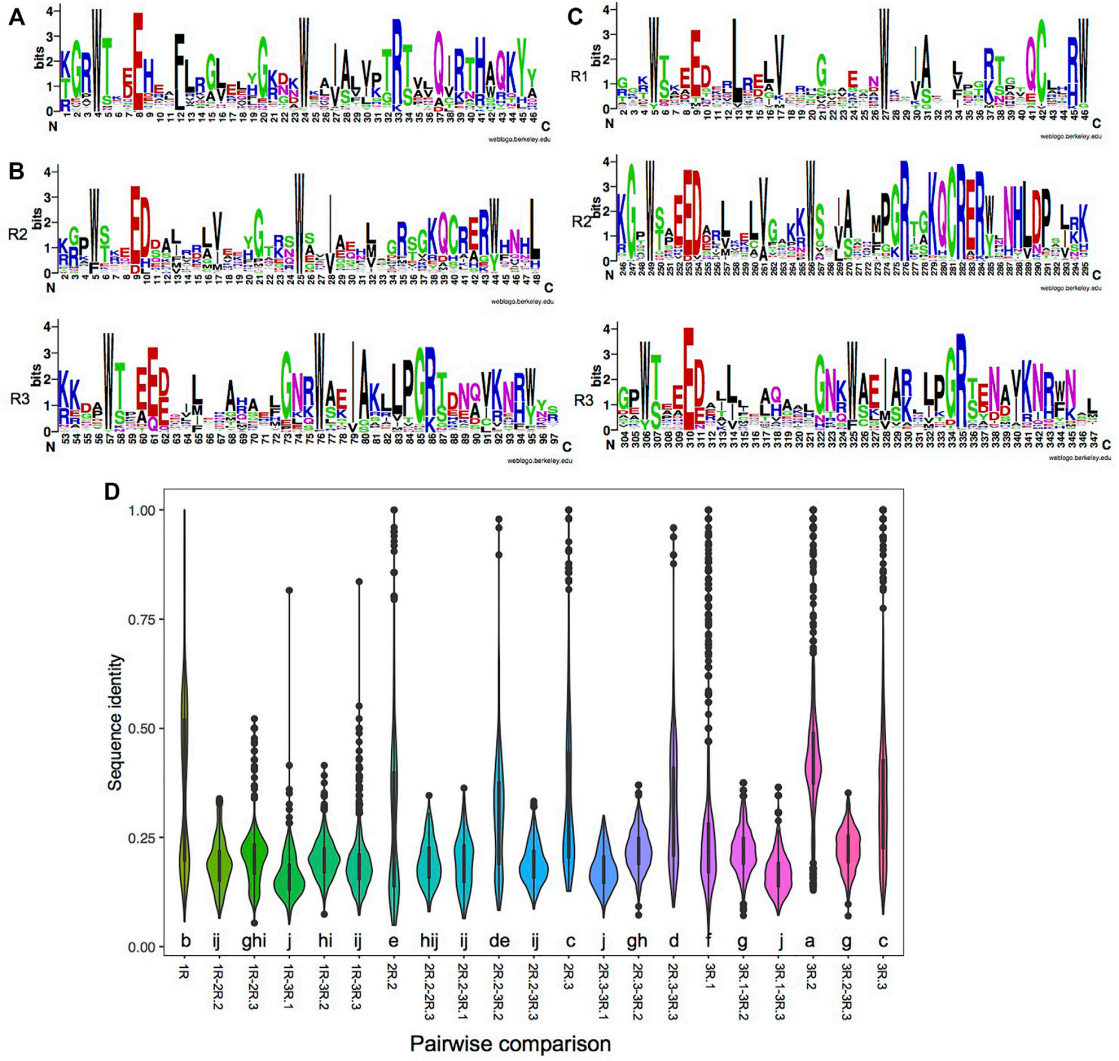
Characteristics of each repeat of MYBs (myeloblastosis). **(A**–**C)** Consensus sequence logos of the single repeat of 1R-MYBs **(A)**; the R2 and R3 repeats of 2R-MYBs **(B)**; and the R1, R2, and R3 repeats of 3R-MYBs **(C)**. **(D)** Distribution of sequence identity within each repeat and pairwise identity between two repeats. The *letter below the violin* indicates the significance level among different comparisons using the least significant difference (LSD) test, *p* < 0.05. The *same letter among groups* represents no significant difference (*p* > 0.05), while *different letters among groups* represent significant difference (*p* < 0.05).

We found that most MYB domains were located at the N-terminal, which is the same as that for MYBs from other plant species. However, the MYB domains were also found in the middle region and in the C-terminal region, i.e., throughout the entire protein sequence of MYB (Supplementary Figures S1–S3). Thus, the location of MYB domains is less conserved in comparison to what was found in plant 1R-MYB (Du et al., 2013). Alignment analysis revealed that the MYB domains are very diverged. While the MYB domains contained the three regularly distributed Trp (W) residues, unusual amino acid substitutions were observed within each repeat, which may influence the specificity of DNA binding. 1R-MYBs are a highly heterogeneous subfamily, and the proteins usually contain other functional domains, reflecting their functional diversity. The first and second W residues were conserved, while the third W was often substituted (Figure 1A and Supplementary Figure S4). The maximum likelihood (ML) phylogeny of 1R-MYB showed that most 1R-MYBs belong to the CCA-like group with strong support value (Supplementary Figure S1). Circadian clock-associated (CCA)-like 1R-MYB is the largest subgroup in plants. It is characterized by the conserved SHAQK(Y/F) in the third helix of the MYB domain (Du et al., 2013). Studies in plants have shown that some SHAQKY MYBs are sequence-specific TFs (Rubio-Somoza et al., 2006). Similarly, brown algae have one such largest subgroup of 1R-MYB. But different from plants, the conserved motif SHAQKY was not found in brown algae. Instead, 22 of the 1R-MYBs belong to the THAQKY subgroup. We also found this motif in the cellular slime molds *Dictyostelium discoideum*, indicating that THAQKY is an ancient motif. Besides, the alanine (A) in this motif was found to be substituted by G, S, and H as well, forming seven THSQKY, four THGQKY, and three THHQKY motifs. Another two single-repeat MYB proteins (SJ09381 and Ec-24_000780) resemble a family of telomeric DNA-binding proteins (TBPs) found in animals and plants, characterized by the consensus motif LKDKWRN (Karamysheva et al., 2004). The existence of these highly conserved motifs in the domain indicates a common origin of the MYB subgroups. Intraspecific tandem duplication was mainly found in the 1R-MYB of *E. siliculosus*, giving rise to Ec-16_000310 and Ec-16-000340, which strongly clustered together. Unlike the diverse domain composition in 1R-MYBs, most 2R-MYBs and 3R-MYBs contain only a DBD. A DnaJ domain (IPR001623) was found in the three brown algae (Supplementary Figure S2) and also existed in the animal species examined, such as fish and mouse. For the R2 of 2R-MYBs, the second W was conserved, while the first and third W were often substituted by F and Y, respectively. For the R3 of 2R-MYBs, the first and second W were conserved, while the third W was often replaced by Y and F (Figure 1B and Supplementary Figure S4). The R1 of 3R-MYBs was less conserved compared with R2 and R3 (Figure 1C) since it does not directly interact with DNA (Feng et al., 2017). The second W of R1 was conserved, while the first and third W were often replaced by Y and G. The first and second W in the R2 of 3R-MYB were conserved, while the third W was often replaced by Y and F. For the R3 of 3R-MYB, all three W were not really conserved. The first and second W were often replaced by Y and F, respectively, while the third W was more heterogeneous, often replaced by F, Y, and other amino acids. Taken together, for all the R repeats, the first and second W were relatively conserved, while the third W was more divergent. The third helix in the MYB domain play key roles in recognizing *cis*-elements in target genes (Du et al., 2013). The conserved W residues function in maintaining the three-dimensional structure of the repeat and forming the hydrophobic core. The specific amino acid in the third helix may reflect their specific recognition sites in target genes.

In a more detailed comparison of the different repeats, we observed 16.2%–43.7% sequence identity among the different kinds of repeats. The highest identity was found in the R2 of 3R-MYBs (43.7%), indicating its conserved amino acid composition. As was expected, higher identity was found between the R2 of 2R-MYBs and 3R-MYBs (30%) and between the R3 of 2R-MYBs and 3R-MYBs (31%) (Figure 1D). In the phylogenetic tree using all repeats, they were also grouped together, further supporting the homology between R2R3 in 2R-MYBs and R2R3 in 3R-MYBs (Supplementary Figure S5). Although similar, there were some differences between the R2R3 domain of 2R-MYBs and 3R-MYBs. For example, D to H and L replacements occurred in the R2 of 2R-MYBs, while D to V replacements occurred in the R2 of 3R-MYBs. The conserved KQCRER motif in the R2 of 3R-MYBs was often replaced by other residues in the R2 of 2R-MYBs. These differences may affect the DNA recognition and interaction with the two repeats and may lead to differences in the DNA recognition specificity between 2R-MYB and 3R-MYB proteins (Williams and Grotewold, 1997). Besides, the R1 of 3R-MYBs was found to have higher identity with the R2 of 3R-MYB (22%), suggesting that the R1 may have come from tandem duplication of R2. The repeat of 1R-MYBs showed high identity within itself (37%) and formed a large separate group with some R3 repeats (Supplementary Figure S5). Interestingly, it exhibited high identity with the R3 of 2R-MYBs (21%) and the R3 of 3R-MYBs (20%), but showed the lowest identity (16.2%) with the first R of 3R-MYBs, indicating that it likely came from the loss of the first and second repeats of 3R-MYBs.

Structural information on the DBDs of the different MYB family members will aid in understanding their functional specialization (Millard et al., 2019). Three-dimensional structures of MYBs had been solved for viruses and animals (e.g., 1H8A and 1MSE), whereas no plant MYB structure has been solved as far as we know. The structures of brown algal MYB DBDs were inferred using homology modeling with the most similar available sequences from non-plant MYBs. As an example, the DBD of MYB1R (SJ02027) had the highest sequence identity (45%) and coverage (16%) with the Myb-SHAQKYF family in *Entamoeba histolytica* (6nvz.1.A) (Supplementary Figure S6). The DBDs of MYB2R (SJ06574) and MYB3R (SJ06620) were modeled based on the MYB DBD of c-MYB from mouse (1h88.1.C) and, thus, likely bind to DNA in the same way.

### Motif and Gene Structure Evolution

**FIGURE 2:**
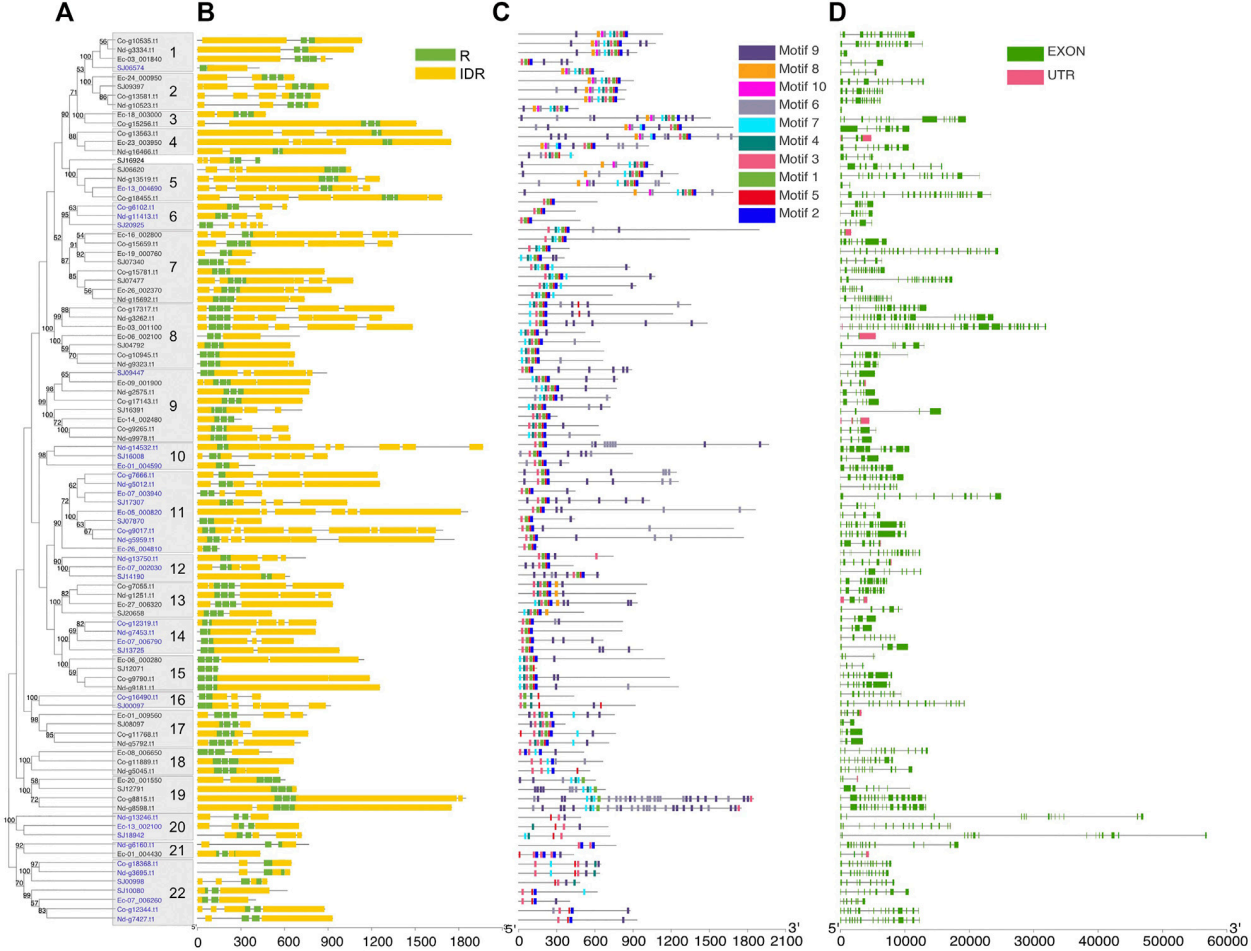
Phylogeny and sequence structure of 2R-MYB and 3R-MYB. **(A)** Tree constructed based on the alignment of the R2 and R3 domains with the maximum likelihood (ML) method using MEGA X with the Le-Gascuel (LG) + gamma (G) model. *Values on the nodes* indicate the percentage of bootstrap support for 1,000 replicates. Bootstrap values higher than 50% are displayed. *Boxes on the branches* show the subgroups with strong support, defined as more than 80%. The *blue color* of the gene ID represents 2R-MYBs; the others are 3R-MYBs. **(B)** Disorder prediction using ESpritz and DNA-binding domains using Pfam. *Green* and *yellow colors* represent repeat (R) and intrinsic disordered region (IDR), respectively. **(C)** The 10 conserved motifs were predicted using the MEME Suite web server. The sequence logo of each motif is shown in Supplementary Figure S7. **(D)** Exon/intron structures of MYB (myeloblastosis) genes. The exon, intron, and UTRs are represented by the *green box*, *gray line*, and *red box*, respectively.

**FIGURE 3:**
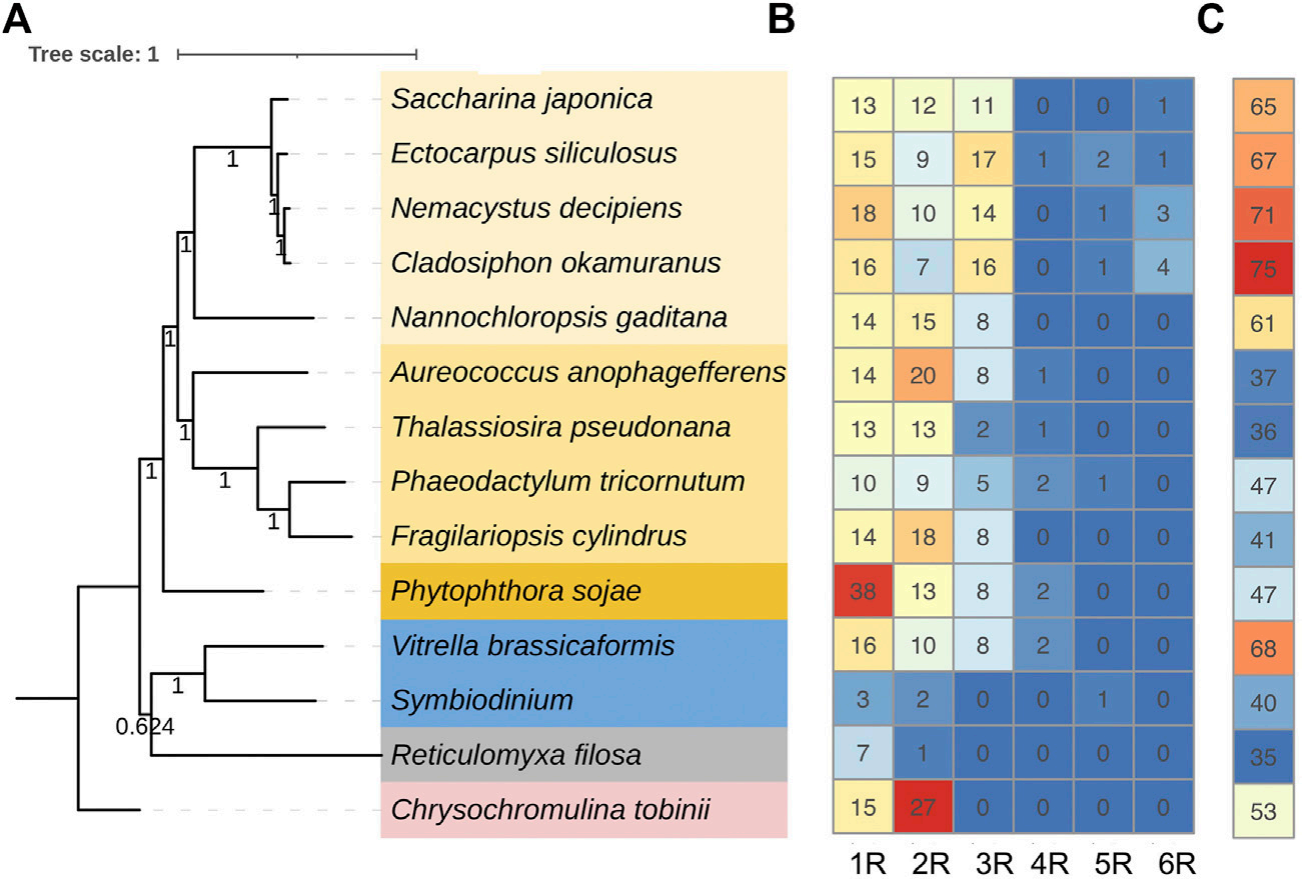
Number of each type MYB (myeloblastosis) and the average intrinsic disordered region (IDR) percentages found in representative species. **(A)** Species tree of the representative species in stramenopiles (*yellow background*), alveolates (*blue background*), rhizarians (*gray background*), and haptophytes (*pink background*). The tree was constructed using the 29 single-copy genes of the 14 species. **(B)** Number of each type of MYB found in each species. **(C)** IDR percentage of the MYB proteins in each species. IDR was predicted using ESpritz.

To further understand the structure–function relationships of brown algal MYB TFs, we collected the dataset comprising domain information (from the Protein Families database, Pfam), structural disorder prediction (from Espritz in the Database of Disordered Protein Prediction, D^2^P^2^), and the location of conserved motifs (from Multiple Expectation Maximization for Motif Elicitation, MEME) for the brown algal MYBs and plotted the information onto the phylogenetic tree (Figure 2). Motifs outside the MYB domain are of variable lengths and low sequence conservation (Stracke et al., 2001; Dubos et al., 2010) and are significant signatures of closely related MYB members (Jiang and Rao, 2020). We found that the non-MYB regions had higher sequence diversity and extensive intrinsically disordered regions (IDRs; Figure 2B). They might be important to the high functional diversity of brown algal MYBs. Almost all of the sequences had IDRs longer than 30 amino acids. The average percentages of IDRs in the MYB proteins of the four brown algae were 65%, 67%, 71%, and 75%, respectively (Figure 3). Generally, the N-terminal DBD is followed by a large IDR at the C-terminal, although many subgroups have N-terminal disordered extensions, such as subgroups 1–5. Overall, the non-MYB regions vary in length and generally contain large disordered segments. We searched for 10 conserved motifs throughout the protein sequences. MYB proteins within the same clade usually have similar compositions of motifs, but variations were observed among different clades, which has aided phylogenetic assignment. For 1R-MYBs, motifs 2, 7, and 1 corresponded to the first and second W and THAQK, respectively (Supplementary Figures S7, S8). Besides them, only very few motifs were found in each sequence, suggesting a high divergence outside of the MYB domains, although a few contained features typical of transactivation domains (Latchman 2010). These transactivation domains included low-complexity regions with amino acid compositional bias in motifs 3 and 8, which were composed of multiple glutamines (Q). For 2R-MYBs and 3R-MYBs, among the 10 motifs, motifs 1, 2, 3, 4, 7, and 8 contained W, while motifs 6 and 9 were multiple Q and A, respectively (Figure 2C and Supplementary Figure S9). From the tree and motif composition, we can see that 2R-MYBs mainly comprised motifs 3, 1, 5, and 2, while 3R-MYB had motifs 7, 3, 1, 5, and 2, which composed the R1, R2, and R3 domains. Unlike 1R-MYB, 2R-MYB and 3R-MYB had not only multiple Q in motif 6 but also multiple A in motif 9. Interestingly, some MYB proteins contained the repetition of motifs 6 and 9, indicating their involvement in some specific functions. Overall, the motif organization of proteins within the same subgroup in the phylogenetic tree was relatively conserved, indicating that they have a common origin and may be involved in the same or similar biological functions. The conserved motif may be essential for MYB function. It was suggested that the motifs of MYB may be involved in protein–protein interactions, posttranslational modifications, or transcriptional activation or repression (Millard et al., 2019). Losses and gains of auxiliary motifs during plant evolution are common in 2R-MYBs and could lead to functional shifts (Finet et al., 2013). However, most of the conserved motifs have not yet been linked to specific functions and need further investigation (Millard et al., 2019; Jiang and Rao 2020).

Almost all of the MYB genes (except five genes in *E. siliculosus*) were disrupted by introns, and the intron count and length varied greatly. The number of introns ranged from 1 to 39, with an average of 5.7 in 1R genes, 8 in 2R genes, and 8.2 in 3R genes. The intron number of 1R genes was significantly lower than that of 2R and 3R genes (*p* < 0.05, Wilcoxon test). On the other hand, the average intron lengths were 1,004, 1,076, and 706, respectively. The intron length of 3R genes was significantly lower than that of 1R and 2R genes (Supplementary Figure S10). Genes with a similar gene structure usually clustered into the same subgroup (Figure 2D). The different intron counts and lengths of the three MYB subfamilies further supported the separated evolutionary history.

### Phylogenetic Analysis

**FIGURE 4:**
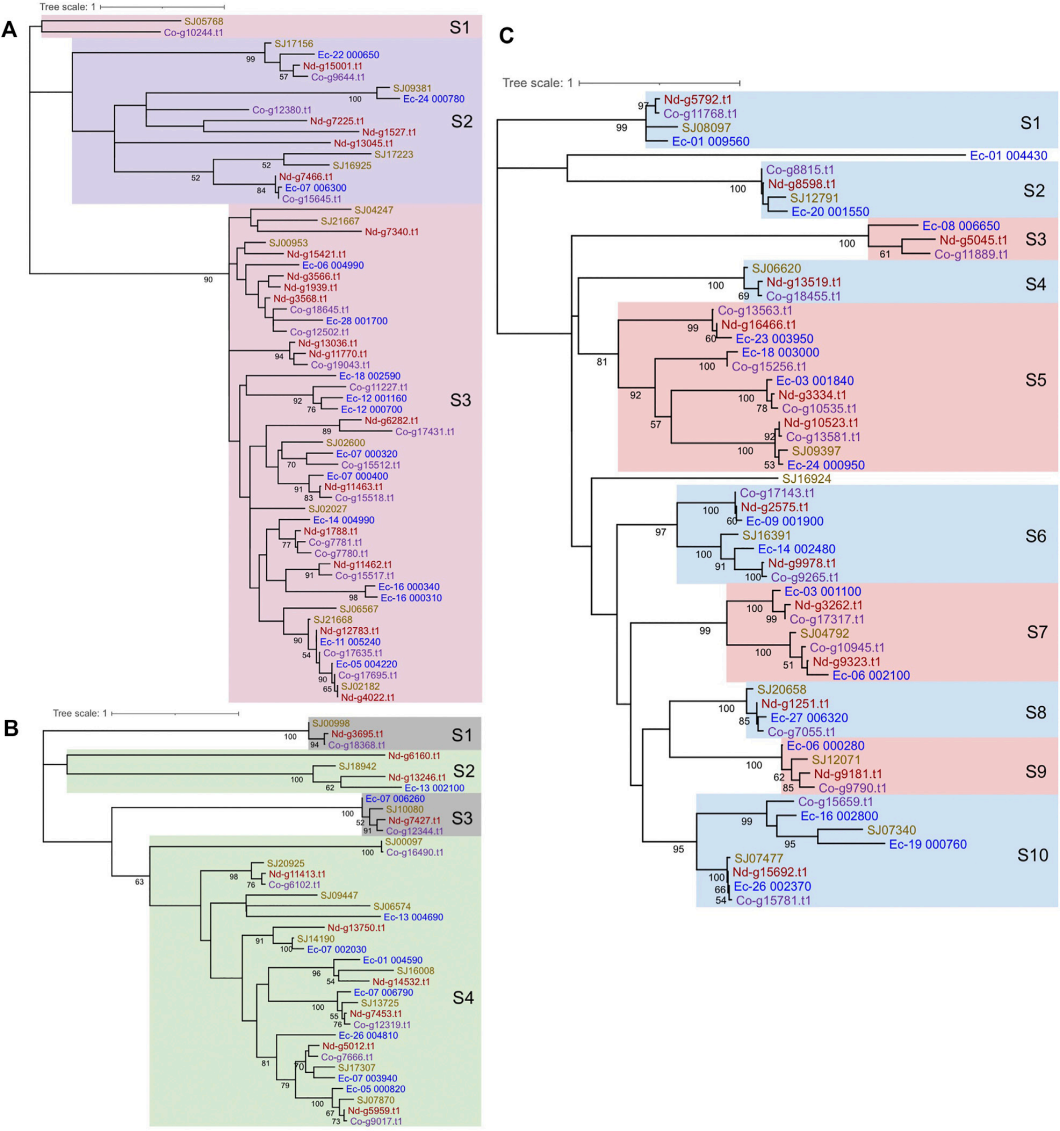
Phylogenetic trees and subgroup classification for brown algal 1R-MYBs **(A)**, 2R-MYBs **(B)**, and 3R-MYBs **(C)**. The tree was constructed with the maximum likelihood (ML) method using MEGA X with the Le-Gascuel (LG) + gamma (G) model based on the alignment of the DNA-binding domains (DBDs). *Support values on the node* indicate the proportion of recovered nodes out of 1,000 bootstrap replicates. Values higher than 50% are displayed. The *same color of gene ID* represents the same species. *Nd* and *Co* in the gene ID represent *Nemacystus decipiens* and *Cladosiphon okamuranus*, respectively.

To determine the phylogenetic relationships among the MYB proteins, phylogenetic trees were constructed. 1R-MYBs were divided into three subgroups, among which TH(A/H/S/G))QKY formed the largest clade with high bootstrap support, while the other two subgroups, S1 and S2, were outer clade with low bootstrap value, but this was anticipated given the short sequences used (Figure 4A and Supplementary Figure S1). At least ten 2R-MYBs and fifteen 3R-MYBs existed in the common ancestor of brown algae. In contrast with the four subgroups of 2R-MYBs, more subgroups were classified in 3R-MYBs (Figures 4B, C), most of which consisted of the four orthologous genes from the four species. When constructing the tree using R2R3 domain of 2R-MYBs and 3R-MYBs, the two subfamilies were classified into 22 subgroups with strong support, including seven subgroups of 2R-MYBs and 15 subgroups of 3R-MYBs (Figure 2A). Genes within the same subfamily tended to cluster together and separated from the other subfamily, supporting the independent evolution of the two subfamilies, except subgroups 1, 5, 9, and 21, in which one 2R-MYB was grouped with 3R-MYB. This mixture might have resulted from the recent loss of the first repeat in the 3R-MYB. The MYB genes in most of the subgroups were single-copy orthologous genes, in that each subgroup contained only one copy in each species, suggesting that the duplication occurred before the species diverged. On the other hand, two copies of paralogous genes were found in subgroups 7, 8, 9, 11, and 22, in which one duplication event occurred before the species diverged. Interestingly, unlike 1R-MYBs, in which intraspecific tandem duplication was found in *E. siliculosus*, no tandem duplication was found within each species in 2R-MYBs and 3R-MYBs, further supporting their ancient origin.

**FIGURE 5:**
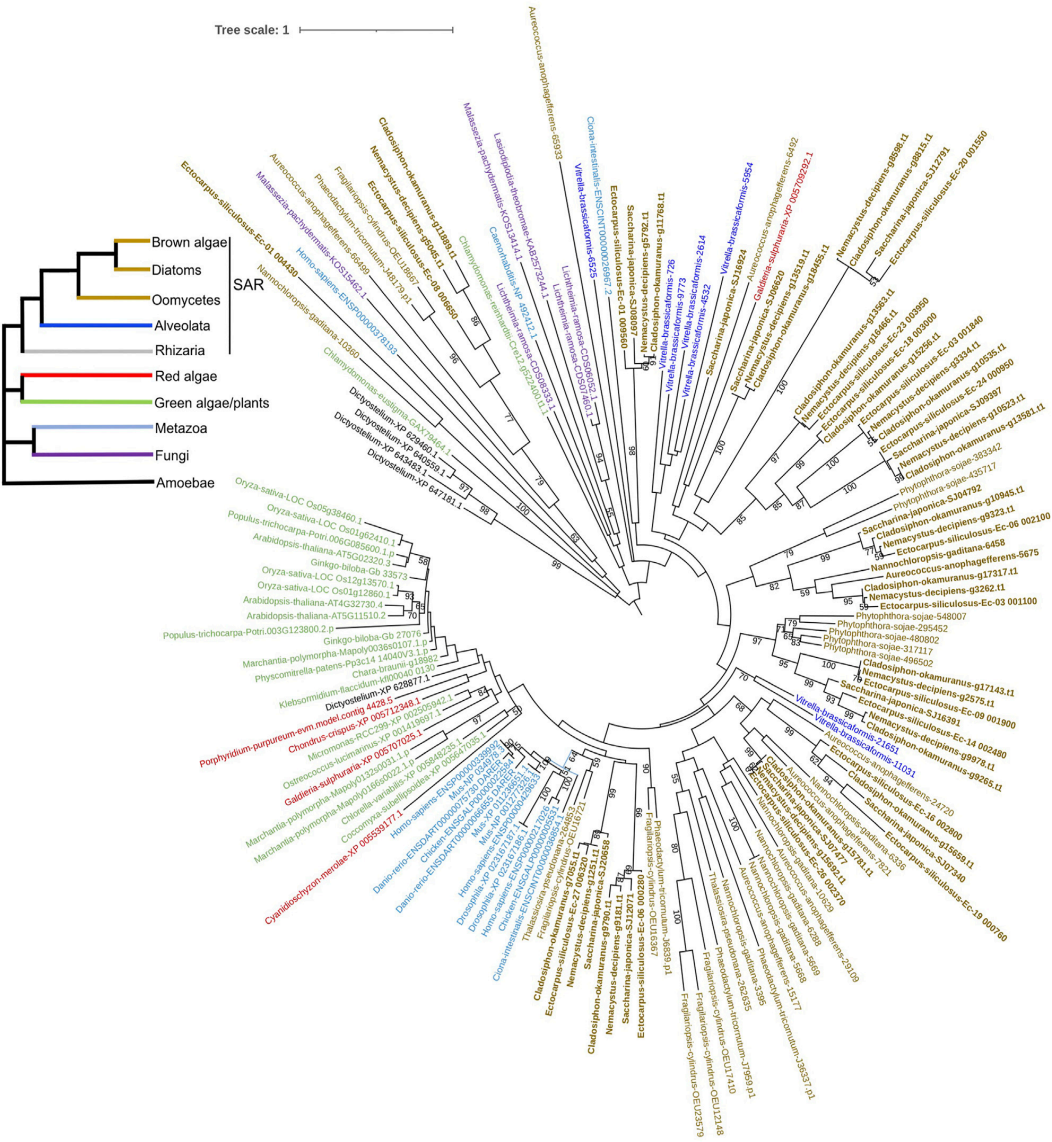
Phylogenetic tree of 3R-MYBs with extended species sampling. The tree was constructed using the IQtree program embedded in PhyloSuite based on the alignment of the R1R2R3 domain using 124 amino acid sites from 156 sequences. The bootstrap was inferred from 500 standard replicates, and support values higher than 50% are shown. *Different branch colors* represent different lineages. The evolutionary relationships of the six major eukaryotic lineages included in the tree, which are represented by *different branch colors*, are illustrated on the reference species tree in the *top left corner*.

To explore the origin of brown algal MYBs, we used MYBs from more species to construct the phylogenetic tree, including animals, fungi, green plants, green algae, red algae, amoebozoa (*D. discoideum*) and other SAR species. Most sequences represented divergence events occurring deep in the tree and had low statistical support. We found that all three subfamilies of brown algal MYBs are not monophyletic; that is, they are distributed throughout different clades and separated by other lineages (Figure 5 and Supplementary Figure S11, S12). Most brown algal MYBs tended to group together with other stramenopile species, while no strong affinity was found with red, green algae, or opisthokonta species, so it is not obvious which lineage is their closest ancestor. It is possible that MYBs existed in the common ancestor of all eukaryotes and diversified independently in each lineage. It should be noted that, compared with the fewer MYB3R in other eukaryotes, brown algae have an expanded MYB3R, which clustered into seven separate groups with other stramenopile species. Interestingly, many stramenopile 3R-MYBs were outside and separated from all other lineages, suggesting that they are ancient and have existed before the origin of plants and animals. We can infer that the duplication of MYB3R in brown algae should have a very ancient history. The retention of a higher number of 3R-MYB copies over long evolutionary periods suggests that they probably have acquired new functions and have been maintained by selection pressure. Further genetic approaches will be necessary to discover the nature of their functional diversity.

### Synteny Analysis and Chromosomal Distribution of MYB Genes

**FIGURE 6:**
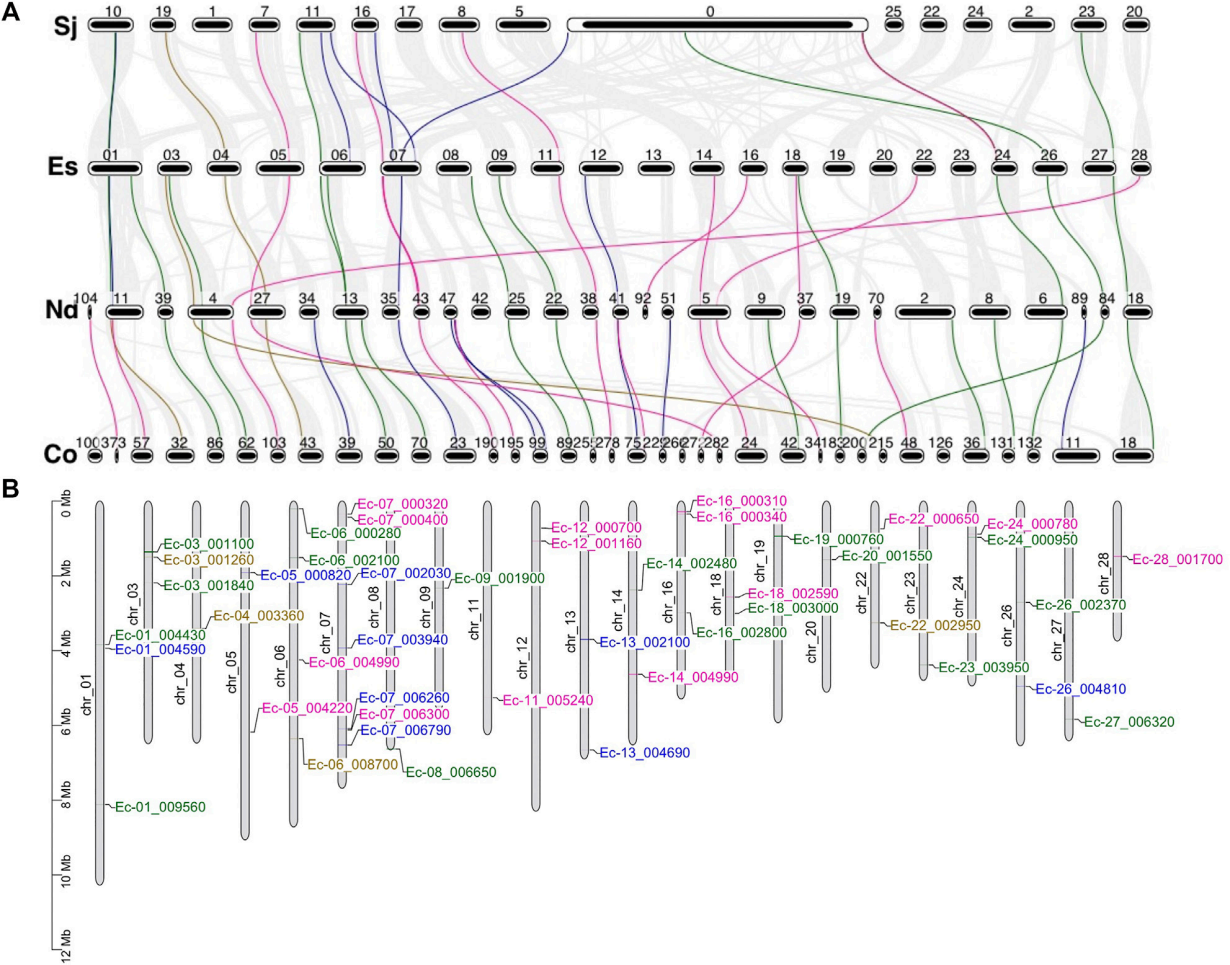
Synteny analysis and genomic distributions of MYB (myeloblastosis) genes. **(A)** Synteny analysis of the MYB genes from the four brown algae. *Numbers* represent the chromosome or scaffold. *Gray line in the background* indicates collinear blocks between the two species, while *colored lines* highlight the syntenic MYB gene pairs. **(B)** Chromosomal location of MYB transcription factors (TFs) in *Ectocarpus siliculosus* [*pink*: MYB1R; *blue*: MYB2R; *green*: MYB3R; *brown*: genes with more than three repeats (R)].

To evaluate the evolution and duplication mechanisms of brown algal MYB genes, comparative synteny maps between the four species were constructed. Orthologous MYB gene pairs were identified to locate in the collinear blocks: 13 between *E. siliculosus* and *S. japonica*, 24 between *E. siliculosus* and *N. decipiens*, and 35 between *N. decipiens* and *C. okamuranus* (Figure 6A). The fewer collinear genes between *E. siliculosus* and *S. japonica* may be due to the large numbers of genes that were not assigned to the true chromosomes in *S. japonica*, and these genes were put into the large pseudochromosome 0. The stronger linear relationship between *N. decipiens* and *C. okamuranus* may be explained by the closer evolutionary relationship between them or the same genome sequencing method applied. At least eight orthologous groups displayed collinearity among all of the four species, including three MYB1R, three MYB3R, one MYB2R, and one MYB6R, suggesting that they have a common origin. Interestingly, three MYB6Rs (SJ07453, Co-g9305.t1, and Nd-g4149.t1) and one MYB5R (Ec-04_003360) existed in one collinear block. This further supports that more than four MYB repeats had already existed before the divergence of the four algae. Besides, we plotted the chromosomal distribution of the MYB genes in *E. siliculosus*, which had assembled high-quality chromosomes (Figure 6B). The analysis revealed that MYB genes were distributed throughout nearly all chromosomes of *E. siliculosus*. Chromosome 7 had seven MYBs, which is the highest number, followed by chromosome 6 with 4 genes. Other chromosomes harbored one to three MYB genes. Tandem duplications were mainly detected in 1R-MYBs, of which three pairs of genes were distributed in tandem and clustered together in the phylogenetic tree. However, none of the 2R or 3R genes were closely linked, suggesting that tandem duplication did not contribute to the 2R-MYB and 3R-MYB expansion and they should arise from the segmental or even genome-wide duplication.

### Expression Analysis

**FIGURE 7:**
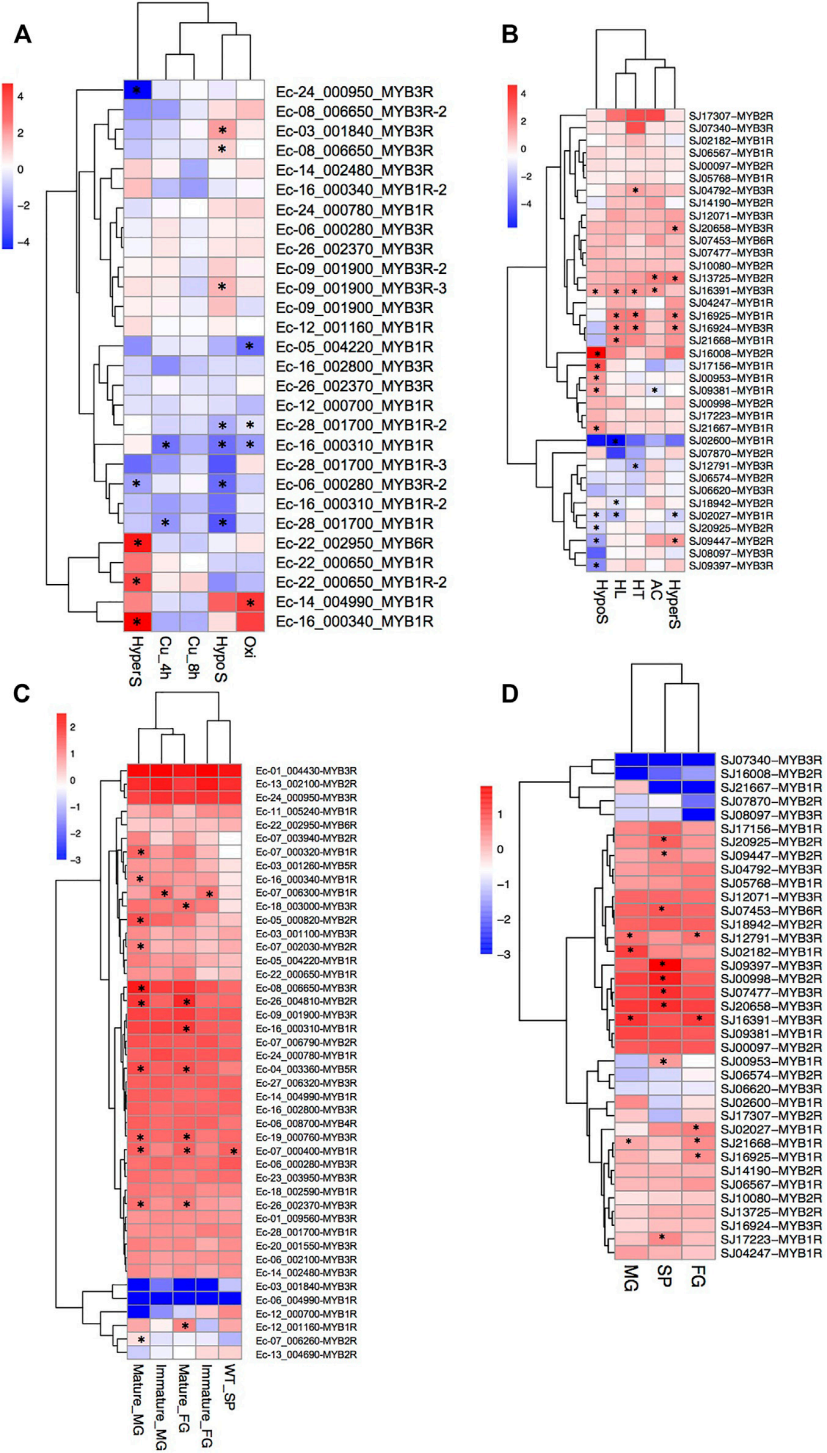
**(A**–**D)** Expression profiles of MYB (myeloblastosis) genes in *Ectocarpus siliculosus* **(A**, **C)** and *Saccharina japonica* **(B**, **D)**. **(A**, **B)** Log2-transformed fold changes of the expression levels compared to the control. *Black star* indicates the significantly differently expressed genes (fold change >2, *p* < 0.05, *t*-test). **(C**, **D)** Log10-transformed RPKM (reads per kilobase of transcript per million mapped reads) values. *Black star* indicates the significantly highly expressed genes compared to the other life stages (fold change >2, adjusted *p* < 0.05, ANOVA). *SP*, sporophytes; *FG*, female gametophytes; *MG*, male gametophytes.

We analyzed the gene expression profiles of the MYB genes in the two brown algae *E. siliculosus* and *S. japonica* (Figure 7). Nineteen MYB genes in *E. siliculosus* were present in the microarray data, and eight of them had two or three contigs/singletons. Hierarchical clustering showed that the expression levels of several MYB genes were profoundly affected by stress conditions. One MYB6R and two MYB1Rs were significantly upregulated, while two MYB3Rs were downregulated under hypersaline stress (fold change > 2 and *p*-value < 0.05). Three MYB3Rs were significantly upregulated, while three MYB1Rs and one MYB3R were downregulated by hyposaline stress. One MYB1R was upregulated, while three MYB1Rs were downregulated by oxidative stress. On the contrary, MYB genes seemed to be less sensitive to copper stress, with only two MYB1Rs downregulated. From the patterns above, we inferred that at least some MYB3Rs should participate in the salt response pathway, in which low salinity activates while high salinity represses MYB3R transcription. On the other hand, MYB1R genes were more sensitive to oxidative stress, with one upregulated and three downregulated. Regarding *S. japonica*, hierarchical clustering showed that more genes were responsive to low salt stress, including six upregulated genes and four downregulated genes. Besides, seven (four upregulated and three downregulated), six (five upregulated and one downregulated), five (four upregulated and one downregulated), and three (two upregulated and one downregulated) MYB genes were significantly influenced by high light, high salinity, high temperature, and acidification, respectively. Notably, one MYB3R (SJ16391) was responsive to all four stress conditions, suggesting that this gene plays crucial roles in stress-responsive networks. Expression divergence was also observed in different life stages. In *E. siliculosus*, many MYB genes were significantly highly expressed in mature male and female gametophytes compared to sporophytes or immature gametophytes (*p* < 0.05, ANOVA). Only one gene (MYB1R: Ec-07_006300) was highly expressed in immature male and female gametophytes, while none was found to be highly expressed in sporophytes compared to gametophytes. Unlike *E. siliculosus*, in *S. japonica*, up to nine MYBs exhibited higher expression levels in sporophytes compared to gametophytes, while three genes were highly expressed in gametophytes compared to sporophytes. Collectively, many MYB genes showed distinct patterns of expression at different life stages.

## Discussion

The MYB family is one of the largest TF families and has been involved in diverse important biological processes. MYBs have been identified in many plant species, but only few of other supergroups were examined. SAR evolved from secondary endosymbiosis and have evolved into nearly half of all eukaryote species. Recently, a “TSAR” supergroup has been proposed, with the addition of telonemids to SAR (Burki et al., 2020). We were interested in the evolution of MYBs in this supergroup. How many MYB types do they have? And where did they come from? What characteristics do they have compared to other lineages? With these questions, we carried out a comprehensive investigation of one of the lineages, brown algae, which belong to stramenopiles and evolved into multicellularity.

### Brown Algae Show Distinct MYB Subfamily Composition

The MYB gene family is one of the largest TF families and plays crucial physiological roles in various organisms. The number of MYB genes varies greatly across different lineages. Plants usually have hundreds of MYB genes, such as 197 in *A. thaliana*, 155 in *Oryza sativa* (Katiyar et al., 2012), 244 in *Glycine max* (Du et al., 2012), 256 in *Prunus persica* (Li et al., 2016), and 253 in *Hedychium coronarium* (Abbas et al., 2021). The expansion of 2R-MYBs contributed to the large number of MYBs in land plants. Interestingly, this scenario did not happen in almost all other lineages. In our survey of MYBs in other phyla, only dozens of MYBs were found. For green and red algae, MYBs were not expanded as well. In the green algae surveyed, there were up to 12, 12, and 2 copies of 1R-MYBs, 2R-MYBs, and 3R-MYBs, respectively. Similarly, there were up to 14, 9, and 2 copies of 1R-MYBs, 2R-MYBs, and 3R-MYBs, respectively, in red algae. In the third version of the animal TF database TFDB3, the number of MYBs in each of the 97 species is around 30 or less. A moderate number of 10–30 MYBs was found in fungi (Verma et al., 2017; Wang et al., 2018; Wang et al., 2020). Compared with these phyla, the situation in brown algae revealed another scenario. The number of MYB genes in brown algae is more than that in animals, as well as more than that in red and green algae. More interestingly, 3R-MYBs accounted for a higher percentage of all the brown algal MYB genes, with more than 10 members in each species. Three 3R-MYBs were found in most vertebrates, while most invertebrate genomes encoded a single 3R-MYB (Lipsick et al., 2001; Davidson et al., 2012; Campanini et al., 2015). In angiosperms, there were fewer than 10 genes encoding 3R-MYB proteins (Feller et al., 2011; Feng et al., 2017), although 11 or 15 appeared occasionally (Saha et al., 2016; Salih et al., 2016). Therefore, we inferred that the 3R-MYB genes should have expanded in the brown algal ancestor. We further explored the status in other SAR lineages. Less than ten 3R-MYBs were found in other species. Nine 3R-MYBs were also reported in one oomycete, *Phytophthora infestans* (Xiang and Judelson 2010). These data suggest that 3R-MYBs might have duplicated before the divergence of SAR, followed by losses in some lineages, or that the ancestor of brown algae evolved more 3R-MYBs than did other members of the SAR lineage. Both the animal and plant 3R-MYBs were found to play a conserved role in cell cycle regulation, cellular proliferation, and differentiation (Ramsay and Gonda 2008). It is likely that the 3R-MYBs in brown algae might also play key roles in these aspects. One major mechanism by which they can be maintained is through neofunctionalization, subfunctionalization, or increased gene dosage (Davidson et al., 2012). Gene duplication could generate raw material for biological innovations (Ohno 1970; Campanini et al., 2015). The resulting paralogs evolved to serve divergent functions and regulatory roles (Lynch and Walsh, 2007; Teng et al., 2017b). Indeed, many 3R-MYBs were found to be upregulated under stress conditions.

Besides the canonical 1R, 2R, and 3R proteins, other multi-domain MYB proteins were also identified, including 4R-MYB, 5R-MYB, and 6R-MYB. Genes encoding 4R-MYB were also present in many plants and showed evolutionary conservation in a broad range of eukaryotes (Stracke et al., 2001; Yanhui et al., 2006; Matus and Arce Johnson, 2008; Dubos et al., 2010; Thiedig et al., 2021). We found that they also existed in some SAR species, suggesting its ancient origin. MYBs containing five repeats seemed sparse, while they were also found in the oomycete *P. infestans* (Xiang and Judelson 2010). Interestingly, as far as we know, we report the first finding of 6R-MYB, which contained doubled R1R2R3 domains. It seemed that the single-copy 6R-MYB arose in the common ancestor of brown algae and was maintained in its descendants. We guessed that it might have certain specific functions in the evolution of brown algae.

### Brown Algal MYBs Have High Percentage of Intrinsic Disordered Regions

A high fraction of eukaryotic genomes encodes proteins with IDRs (Yruela and Contreras-Moreira 2012; Dunker et al., 2015). IDRs are the variable regions without a fixed conformational state and are highly sensitive to changes in the environment. Various studies have shown that sequences outside the DBD regions of TF families contain extensive IDRs, which provide specific and unique molecular functions (Liu et al., 2006; Wright and Dyson 2015; Shammas 2017). IDRs could mediate the dynamic interaction of signaling and regulating proteins with many different partners. The interactions are dynamic in binding and allow rapid dissociation, which is required for regulatory roles (Van Roey et al., 2014). More than 90% of *Arabidopsis* TFs contain IDRs longer than 30 residues, and the TFs were significantly enriched in disorder-promoting residues while substantially depleted in order-promoting residues (Liu et al., 2006). However, there are scarce comparative analyses of intrinsic structural disorders in the MYB family. One survey on the disordered regions of the *Arabidopsis* R2R3 MYB family proposed that structural disorder is imperative for the MYBs executing the broad range of molecular functions (Millard et al., 2019). Some molecular functions of non-MYB regions have been verified in *Arabidopsis* R2R3 MYBs, such as the interactions involved in auxin signal transduction, nuclear translocation, and gibberellic acid signaling (Shin et al., 2007; Qi et al., 2014). Although the functional sites of brown algal MYBs are poorly defined, the non-DBD IDRs should be absolutely crucial for biological functions. To uncover the molecular mechanisms of the regulating activity of brown algal MYBs, more studies are necessary to address the protein structure, disorder, and dynamics of these non-DBD segments.

A strong correlation between TF disorder and organismic complexity was found (Yruela et al., 2017). Disorder predictions showed that 83%–94% of the known TFs in eukaryotes possess extended IDRs, which have been a driving force in the evolution of complex multicellularity (Liu et al., 2006; Minezaki et al., 2006; Babu 2016). The disorder of TF proteins increased in concert with organismic complexity. Brown algae comprise the only multicellular lineage in the SAR supergroup. We explored the proportions of disordered MYB residues of brown algae and other species from SAR. Interestingly, brown algal MYBs showed the overall highest disorder percentage (Figure 3C). If this did not occur by chance, the increasing MYB disorder in brown algae likely was an important factor contributing to the evolution of multicellular complexity, which could have facilitated the innovation of more complex signaling and regulatory pathways in response to cell growth and division. Indeed, IDRs and their alternative splicing and posttranslational modifications have been interpreted as a driving force in the evolution of complex multicellularity (Niklas 2014; Dunker et al., 2015). Another explanation regarding the increase of IDRs considered them as by-products of the increase in generation time in multicellular organisms (Lynch and Walsh 2007). Moreover, brown algal MYB family members, especially the expanded 3R-MYBs, likely play key roles in regulating the cell cycle, cell division, cell differentiation, or cell size, which are key processes in multicellular organisms.

### Independent Origin of the Typical MYB Subfamilies

The origin and order of each MYB subfamily have attracted long and intensive interest from evolutionary biologists. It was assumed that MYBs might have a polyphyletic origin, while DBDs were derived from a common origin (Rosinski and Atchley 1998). Afterwards, 1R-MYBs, 2R-MYBs, 3R-MYBs, and 4R-MYBs were thought as separate groups and evolved independently (Stracke et al., 2001; Yanhui et al., 2006; Dubos et al., 2010). Loss-of-repeat and gain-of-repeat are two opposite hypotheses to explain the evolution of MYB subfamilies. It was proposed that 3R-MYBs were generated from 2R-MYBs by gain of one repeat unit (Jiang et al., 2004). However, more evidence supported the “loss” model that R2R3 MYBs originated from 3R-MYBs by loss of the R1 repeat (Rosinski and Atchley 1998; Kranz et al., 2000; Jiang and Rao 2020). With more sequence data available, we were able to investigate the evolution of the MYB subfamilies. The SAR supergroup has been estimated to comprise up to half of all eukaryote species diversity (del Campo et al., 2014; Burki et al., 2020), but the evolution of MYBs in this supergroup is still unknown. Brown algae are the only large multicellular organisms among the SAR. We found that brown algae harbored the highest diversity of repeat number, ranging from 1R-MYBs to 6R-MYBs. Phylogeny, molecular evolution, and structure analysis allowed assessing the origin and evolutionary relationships among each MYB type. Plants, animals, and brown algae are distantly related lineages. No sequence similarity was observed outside of the repeats between plants and animals (Rosinski and Atchley 1998). We also failed to construct a phylogenetic tree using the full protein sequences due to the numerous heterogeneous sites. Although stramenopiles originated from secondary endosymbiosis events, no strong affinity to animal-like host or endosymbiont similar to ancestral red and green algae was detected in the phylogenetic analysis of 1-MYB, 2-MYB, and 3-MYB. Instead, brown algal MYBs showed paraphyletic distribution across the eukaryotic tree. We hypothesized that 1R-MYB, 2R-MYB, and 3R-MYB coexisted in primitive eukaryotes and evolved into extant MYBs in each lineage (Jiang et al., 2004). The R domain of 1R-MYB showed higher identity with the R3 repeat, corresponding to the alternative name of the R3-MYB type proteins (Du et al., 2013). Through sequence comparison among all repeats, we favored the “loss” model that R2R3-MYB originated from 3R-MYB by loss of the R1 repeat and that 1R-MYB originated from the loss of R1R2 of 3R-MYB. Besides, the origin of introns is a controversial topic. The hypotheses of intron gain and intron loss are two opposite opinions (Koonin 2006; Rogozin et al., 2012). Plant 3R-MYBs gained introns stepwise during evolution (Feng et al., 2017). The introns in fungi are short, with mean intron lengths ranging from 69 to 256 bp. The average intron length in *Ganoderma* MYB is 74 bp (Wang et al., 2020). The intron lengths in brown algae are higher. Many MYB genes in brown algae have introns longer than 1,000 bp. If the intron gain hypothesis is valid, more introns in brown algae might indicate the longer evolutionary history. The higher intron count and the lower intron length of 3R-MYBs further support the independent evolution history. In addition, despite the separated evolution, we found that some 2R-MYBs are orthologous to 3R-MYBs and clustered together robustly, which was also found in plants (Jiang and Rao 2020), indicating that the number of MYB repeats in 3R-MYBs may change during evolution. The 2R-MYBs in oomycete clustered with 3R-MYB lineages instead of plant 2R-MYBs, suggesting that the loss of the R1 domain in oomycetes is independent of its loss from the plant lineage (Xiang and Judelson 2010). Likewise, when we constructed the tree using 2R-MYBs and 3R-MYBs involving more eukaryotes, no strong affinity was found in the 2R-MYBs between brown algae and plants (Supplementary Figure S13). Moreover, the DNA recognition sequence of R2 repeat is crucial for DNA binding specificity (Williams and Grotewold 1997). Brown algal 2R-MYBs share the recognition sequence of the R2 repeat (QCRERW) (Myrset et al., 1993) with the R2 repeat of the 3R-MYBs in brown algae, animals, plants, and slime mold, but differ from the corresponding 2R consensus (SCRLRW) in plants, supporting its different evolution from plants, while more similarity with the R2 repeat of animals and cellular slime mold.

## Materials and Methods

### Sequence Data

The most up-to-date genomes of four brown algae (*E. siliculosus*, *S. japonica*, *C. okamuranus*, and *N. decipiens*) were retrieved from public databases. The genome sequences of *E. siliculosus* 2016 version were downloaded from the website http://bioinformatics.psb.ugent.be/orcae/overview/Ectsi (Cormier et al., 2016). The sequences of *C. okamuranus* and *N. decipiens* were downloaded from http://marinegenomics.oist.jp/algae/ (Nishitsuji et al., 2016; Nishitsuji et al., 2019). The sequences of *S. japonica* were downloaded from NCBI https://www.ncbi.nlm.nih.gov/. Other genomes used in this study were downloaded from NCBI, JGI, or the ENSEMBL database. Some MYBs were also acquired from the animal TFDB3 (http://bioinfo.life.hust.edu.cn/AnimalTFDB/#!/) (Hu et al., 2019).

### Identification of MYBs

The MYB genes of brown algae were identified as follows. Firstly, the MYB DNA-binding domain PF00249 was downloaded from the Pfam website (http://pfam.xfam.org/) (Mistry et al., 2021). Then, HMMER3 (Eddy 2011) software with default parameters was used to search MYB proteins in the proteome of each species using PF00249 as a query. The acquired proteins were then used as query sequences for BLASTp searches against the proteome sequences. Redundant sequences were discarded from the dataset to obtain unique MYB proteins. The candidate MYB proteins were then examined using the online InterProScan program (http://www.ebi.ac.uk/interpro/search/sequence-search) to confirm that the sequences have one or more adjacent R repeats and to classify the MYB subfamilies according to the repeat number. Proteins with incomplete or distantly spaced repeats were discarded and not included in further analysis. Besides, the MYBs from green algae, red algae, animals, fungi, plants, and other SAR species were searched and classified using the same procedure.

### Multiple Sequence Alignment and Phylogenetic Analysis

Because of the large sequence divergence outside the MYB domain, we only used the DBDs to construct the phylogenetic trees. For each MYB subfamily, i.e., 1R-MYB, 2R-MYB, and 3R-MYB, DBD sequences were extracted and aligned using both ClustalW and MUSCLE embedded in MEGA X; we chose the better alignment by eye to see which gave a less gappy alignment and a better reconstruction of the conserved MYB domain. ML trees were constructed based on the DBD region alignment with the Le-Gascuel (LG) + gamma (G) model, predicted as the best model by the “Find best DNA/protein models” module of MEGA X. Bootstrap with 1,000 replicates was performed to obtain the confidence support value. In order to trace the origin of brown algal MYB in a much broader context, phylogenetic trees including representative eukaryotic groups, i.e., green algae, red algae, plants, fungi, animals, SAR species, and cellular slime mold, were constructed. Their DBD sequences were aligned using the MAFFT in PhyloSuite (Zhang et al., 2020), and then the alignments were trimmed under “gappyout” mode. The modelfinder in PhyloSuite was used to search the model used for IQtree based on the trimmed alignment. Then, the models were imported into IQtree with 500 standard bootstrap replicates.

### Synteny Block Identification

In order to investigate the origin of MYB genes, we analyzed the synteny blocks among the four brown algal genomes. The genomic neighborhoods surrounding the MYB genes were aligned to each other using the all-to-all blastp method. The blastp results and the combined GFF file were supplied to MCScanX (Wang et al., 2012). The collinearity file generated by MCScanX was used as an input into TBtools to display the collinear relationship, with the MYB genes highlighted (Chen et al., 2020).

### Sequence Structure Analysis

The DBD sequences of each MYB subfamily were aligned using the ClustalW program in MEGA X. Sequence logos for each repeat, R1, R2, and R3 in MYBs were generated using the online WebLogo program (http://weblogo.threeplusone.com/create.cgi) (Crooks et al., 2004). Further detailed DBD alignments were generated using DNAMAN. Intron and exon data of the MYB genes were extracted from the gff3 files of the four brown algae. The conserved motifs of MYB proteins were identified using the MEME program (http://meme-suite.org/) (Bailey et al., 2009). The motif number was set to 10, while the width of the motif was set to range from 10 to 50. The phylogenetic tree of MYB, together with the GFF and xml files of the motif, was input into the TBtools to display the gene structure and motif composition. Besides, for each MYB protein, subcellular protein localization was predicted using Euk-mPLoc 2.0 (http://www.csbio.sjtu.edu.cn/bioinf/euk-multi-2/) (Chou and Shen 2010). The molecular weight and isoelectric point were calculated with the ProtParam tool (https://web.expasy.org/protparam/). Protein transmembrane helices were predicted using TMHMM server 2.0 (http://www.cbs.dtu.dk/services/TMHMM/). Disorder prediction was performed using ESpritz (Walsh et al., 2012). To explore the similarity of the different repeats, all the repeats were aligned and the pairwise sequence identity was generated with BioEdit (Hall et al., 2011).

### Expression of MYB Genes in *E. siliculosus* and *S. japonica*

The expression patterns of MYB genes were examined using the available transcriptome data of *E. siliculosus* and *S. japonica*. We examined MYB gene expression under different life cycle stages and various abiotic stresses. In *E. siliculosus*, we compared the RNA sequencing (RNA-seq) data of parthenosporophytes, male gametophytes, and female gametophytes (Lipinska et al., 2015). Furthermore, previous microarray data of the *E. siliculosus* transcriptome (Dittami et al., 2009; Ritter et al., 2014) were used to explore the expression levels of MYB genes in response to abiotic stresses, including copper stress, hyposaline stress, hypersaline stress, and oxidative stress. The expression level was determined by averaging the expression values (previously quantile normalized by Roche NimbleGen, Madison, WI, USA) of four replicates for each experimental condition. The expression levels of MYB in three life stages of *S. japonica*, i.e., sporophytes, male gametophytes, and female gametophytes, were examined using the RNA-seq data, with three biological replicates of each life stage (Teng et al., 2017a). The stress responses of MYB in *S. japonica* were explored using digital gene expression (DGE) library sequencing (Zhang et al., 2021). Briefly, six RNA samples including control, high light, high temperature, acidification, and hyposaline and hypersaline conditions were used to prepare DGE sequencing libraries and then were sequenced on an Illumina HiSeq 2000 platform. Clean data after quality control were used to calculate the RPKM (reads per kilobase of transcript per million mapped reads) of each gene. Differential expression analysis was performed using the DEGSeq R package. Genes with an adjusted *p*-value <0.05 and a log2(fold change) >1 were considered as significantly differentially expressed compared to the control. A hierarchical cluster was created using the R package.

## Conclusion

In this study, we combined phylogenetic, structural, and gene expression analyses of the MYBs in brown algae to explore the evolution of the brown algal MYB gene family. A total of 172 MYB genes were identified in four brown algae, including 1R-MYBs, 2R-MYBs, 3R-MYBs, 4R-MYBs, 5R-MYBs, and 6R-MYBs. 1R-MYBs and 3R-MYBs were more prevalent compared to 2R-MYBs. Genes with similar numbers of introns and motifs usually clustered into the same subgroup, implying the coevolution of the gene and protein structures. Sequence analysis supports that the 2R-MYB proteins were derived from 3R-MYBs *via* the loss of the first MYB repeat. The repeat of 1R-MYBs showed higher identity with the R3 repeat of 2R-MYBs and 3R-MYBs, raising the possibility that 1R-MYBs came from the loss of the first and second repeats of 3R-MYBs. By incorporating data from other representative eukaryotic organisms, the origin of MYB in SAR could be traced back to the last common ancestor of all extant eukaryotes. MYBs in brown algae are responsive to different abiotic stress conditions and during different developmental stages. This research provides a key reference for the evolutionary and functional investigations of MYB genes in SAR lineages.

## Data Availability

The original contributions presented in the study are included in the article/Supplementary Material. Further inquiries can be directed to the corresponding author.
