# Supplementary material for: Independent Evolution of the MYB Family in Brown Algae
Source: Front Genet. 2022 Feb 4;12:811993. doi: 10.3389/fgene.2021.811993 (PMC8854648; doi:10.3389/fgene.2021.811993)
Supplement: Supplementary file 1 [file DataSheet1.docx]

Supplementary Material

# Supplementary Figures


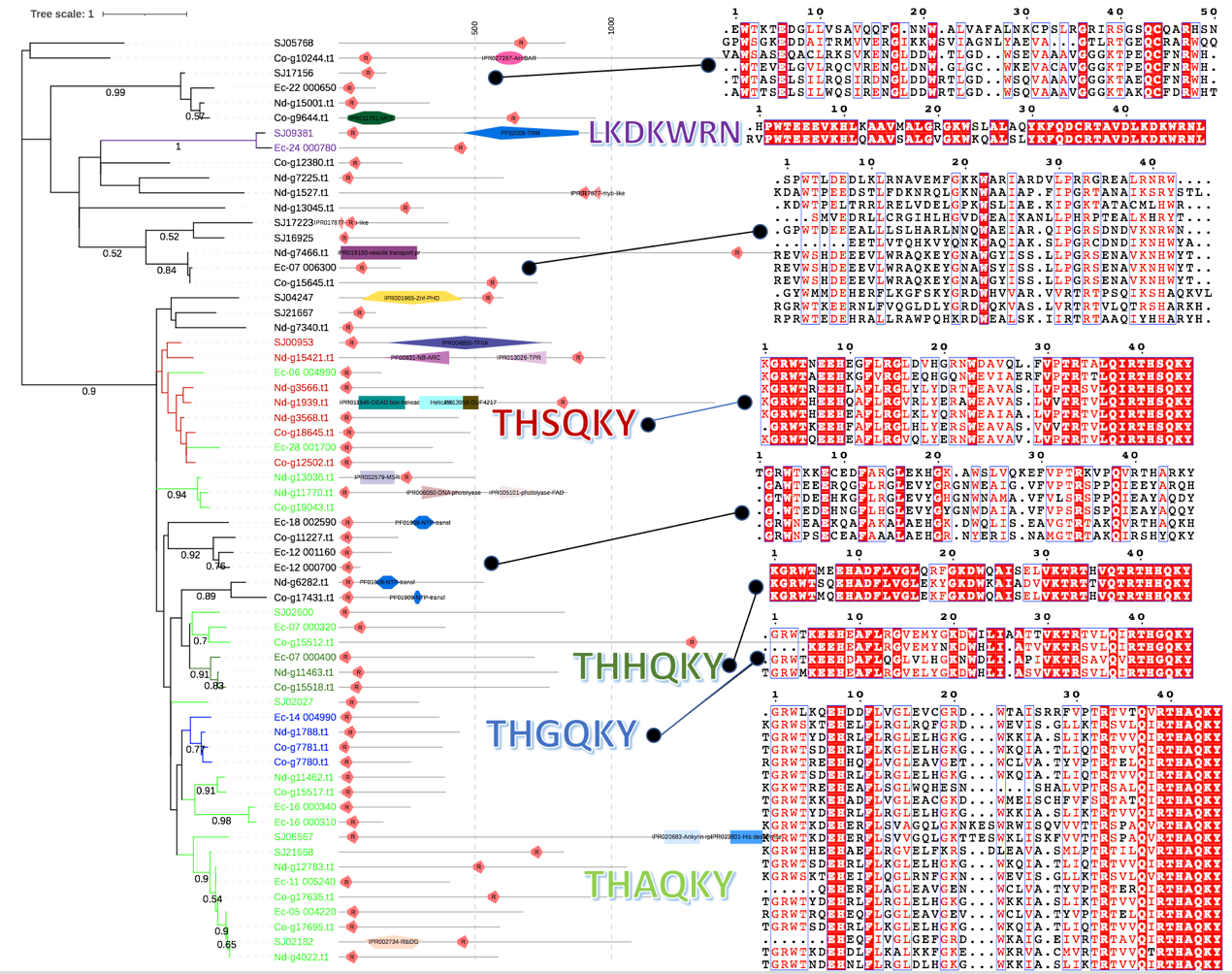


**Supporting information figure S1.** The phylogenetic tree and domain composition of 1R-MYBs. The tree was constructed by ML method using MEGA X with LG+G model based on the alignment of the 1R domain. Support values on the nodes correspond to the relative proportions out of 1000 bootstrap replicates. Support values higher than 50% (0.5) are displayed. The DBD domain and other domains were represented with different shapes. DBD domain is in pink, with the letter R in it. The names of other domains are written in each shape. The color of the gene name represents different motif composition. The corresponding motif was displayed on the right using the letter with the same color, followed by the corresponding sequence alignment. Genes with no consensus motif were in black color and aligned on the right.


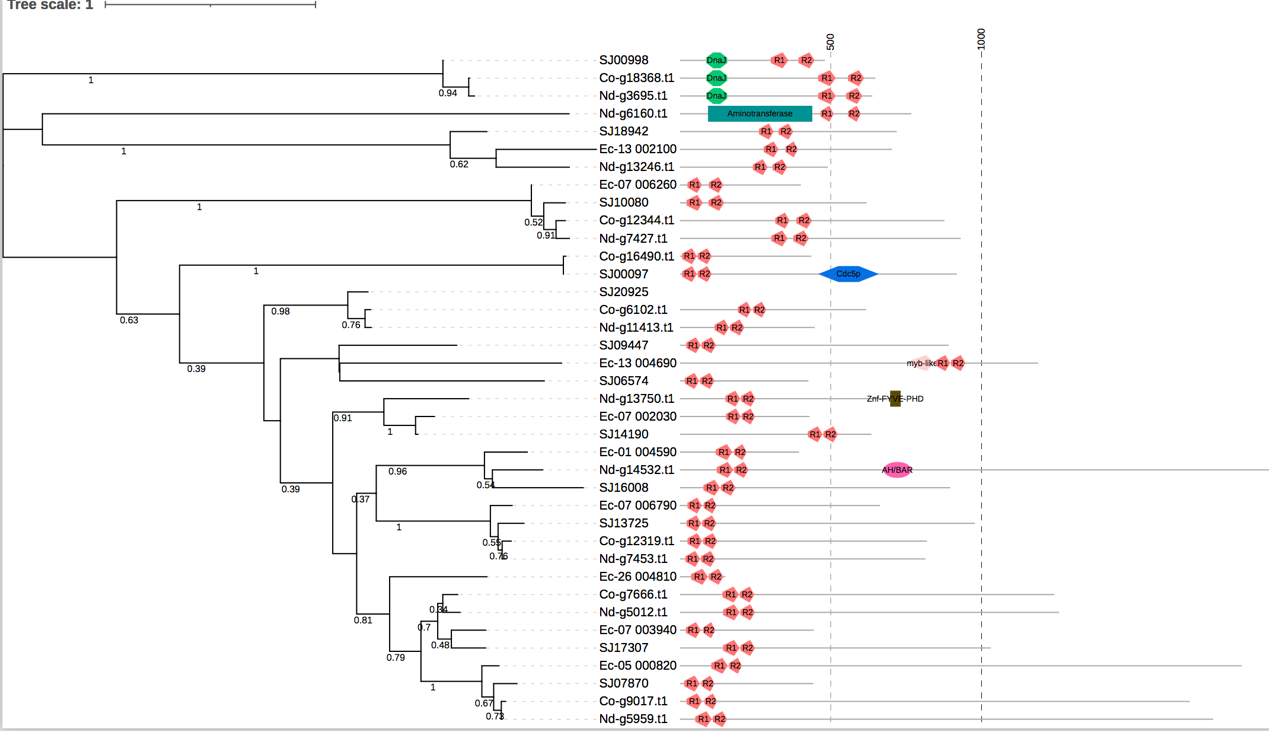


**Supplementary figure S2**. Phylogenetic tree and domain composition of 2R-MYBs. The tree was constructed by ML method using MEGA X with LG+G model based on the alignment of R2R3. The alignment of R2R3 domains was shown in Supporting information figure S4. Support values on the nodes correspond to a proportion out of 1000 bootstrap replicates. The DBD domain and other domains were represented with different shape.


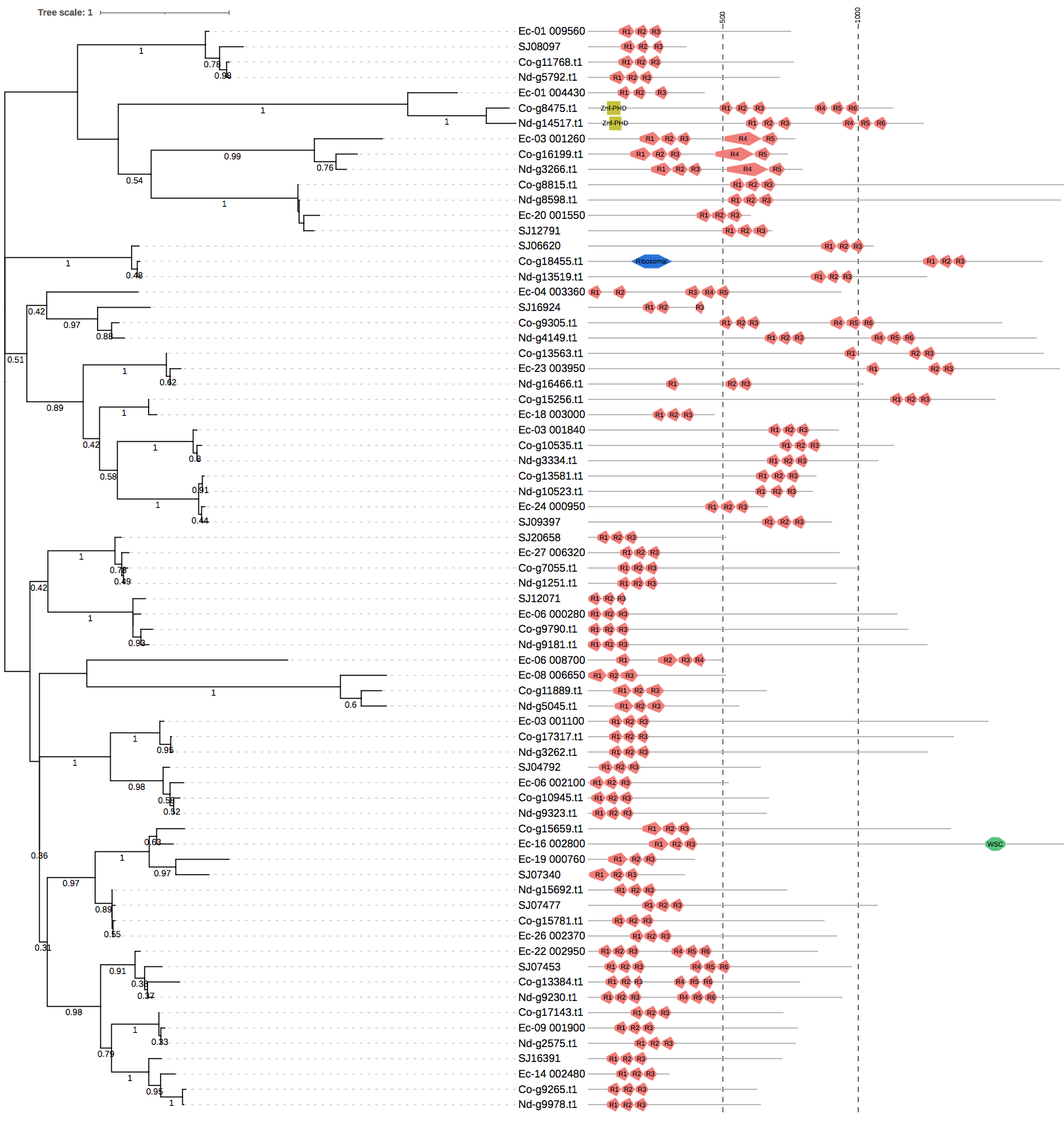


**Supplementary figure S3**. Phylogenetic tree and domain composition of 3R-, 4R-, 5R- and 6R-MYBs. For the MYBs having more than three repeats, the first three adjacent repeats were used for the alignment. The alignment was shown in Supporting information Figure S4. The tree was constructed by ML method using MEGA X with LG+G model based on the alignment of R1R2R3 domain. Support values on the node correspond to a proportion out of 1000 bootstrap replicates. The DBD domain and other domains were represented with different shapes.


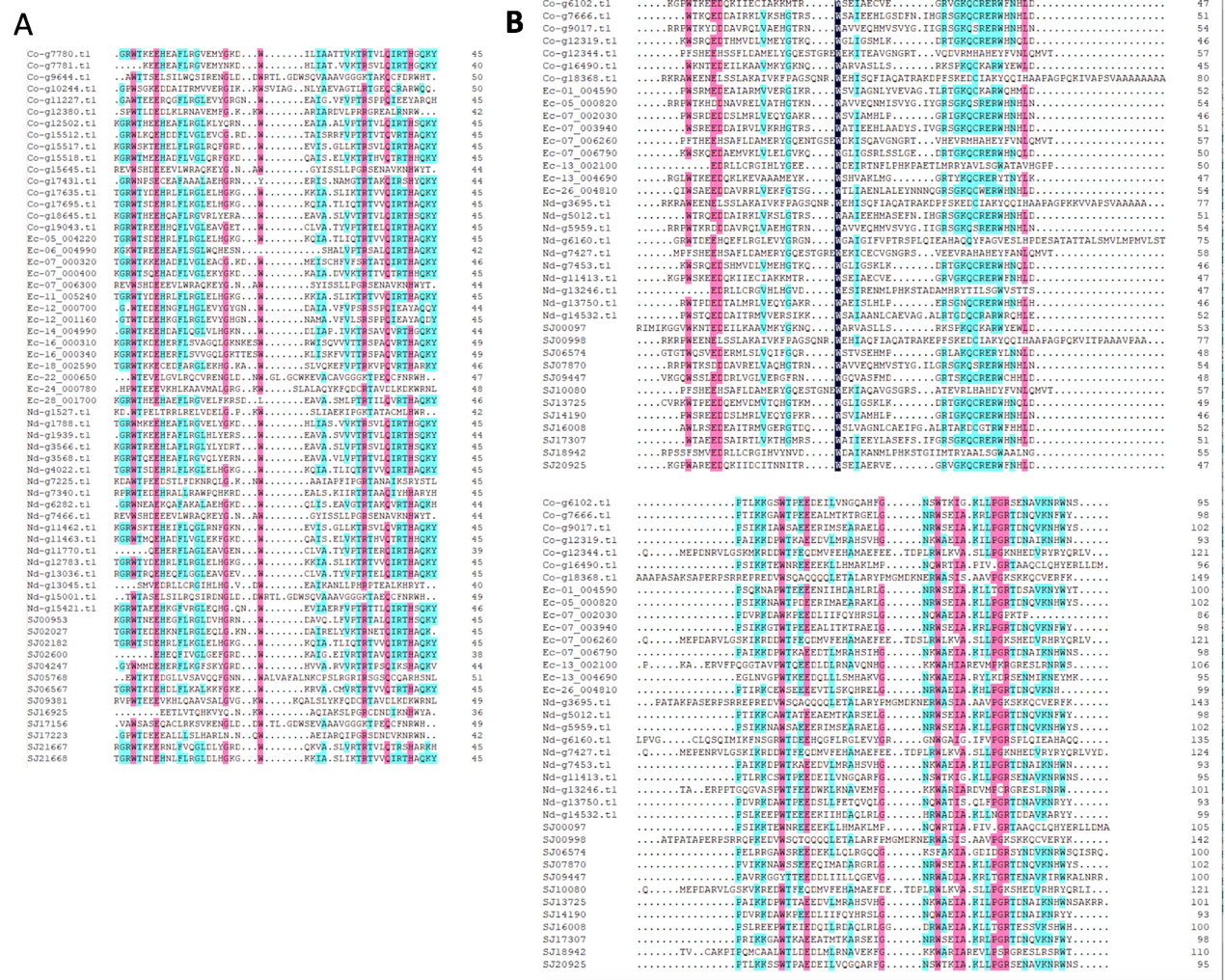


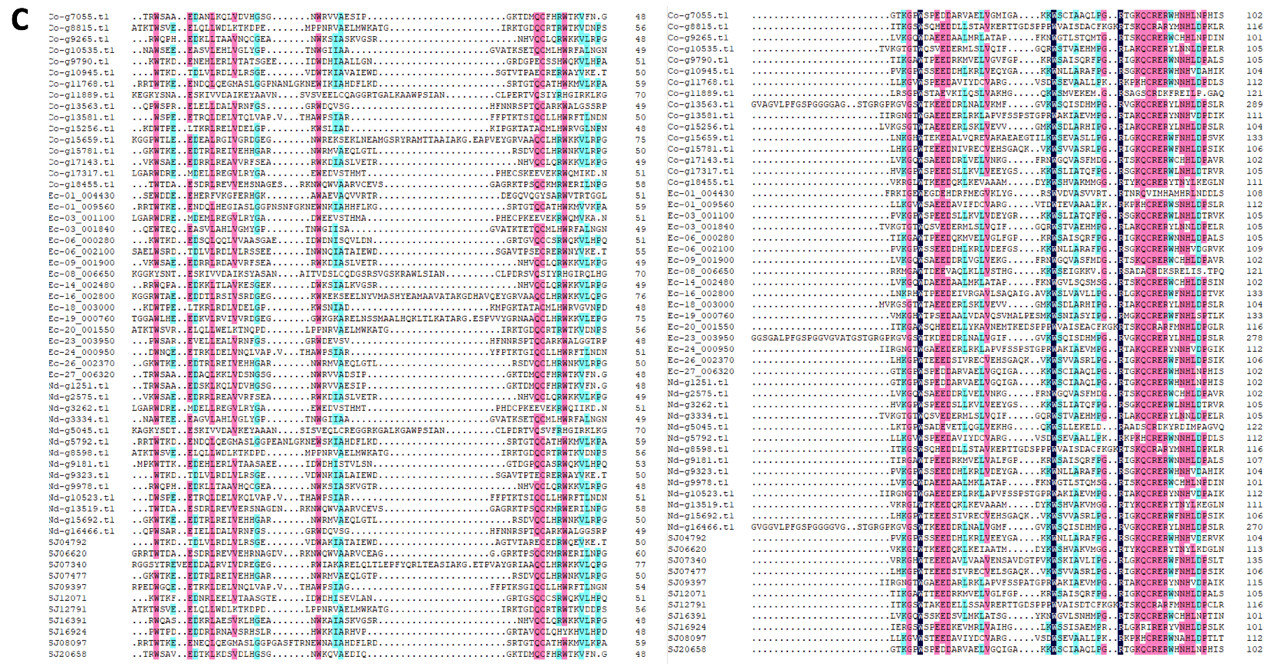


**Supplementary figure S4.** Alignment of DBD domain A. 1R-MYBs; B. 2R-MYBs; C. 3R-MYBs


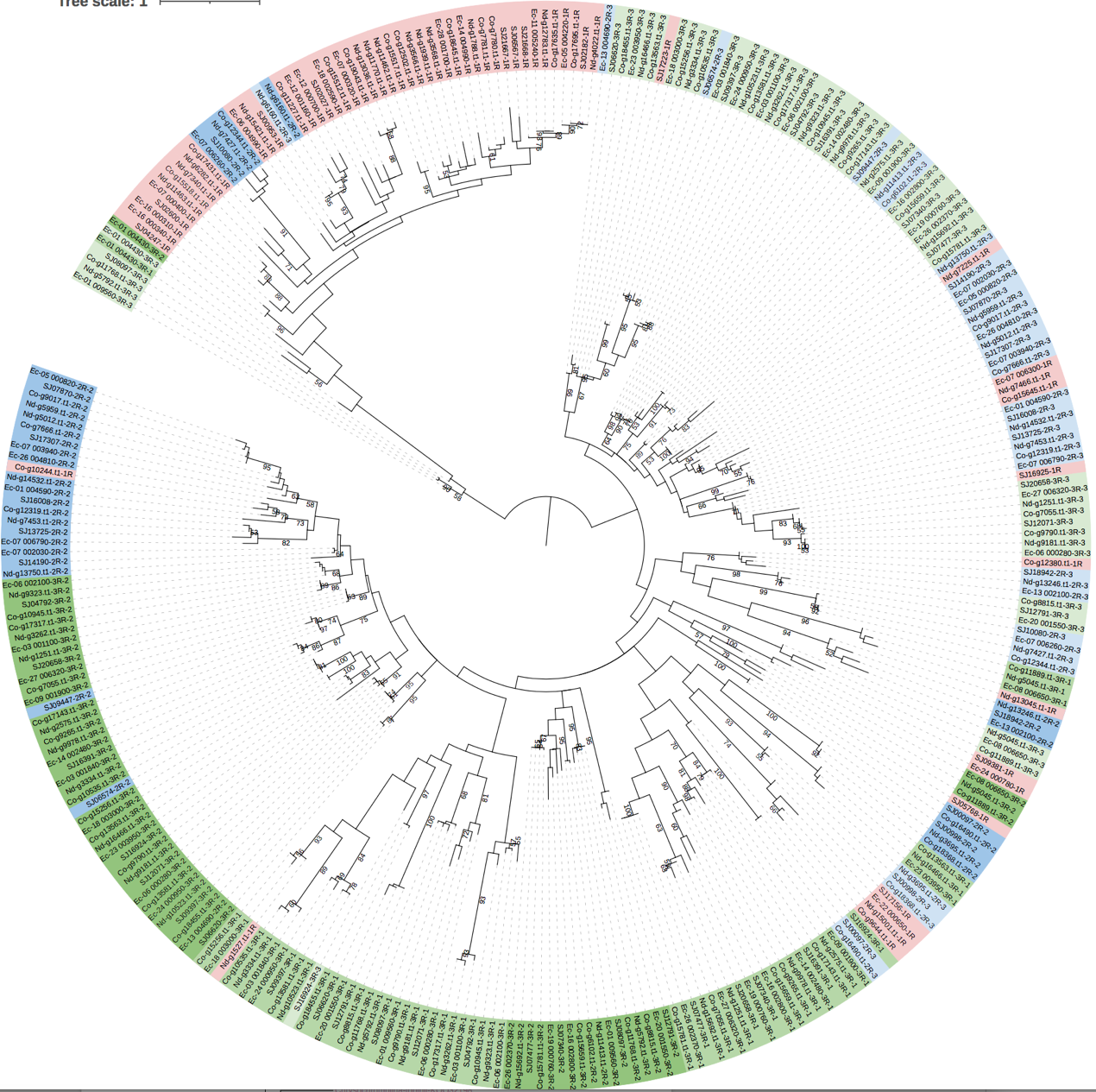


**Supplementary figure S5.** Phylogenetic tree of all separated repeats of brown algal MYBs. The tree was constructed using the alignment of 44 amino acid sites from 304 sequences. Bootstrap support values higher than 50% are displayed. The different background color represents different repeats. Pink, blue, and green color represent the repeat of MYB1R, MYB2R and MYB3R, respectively.


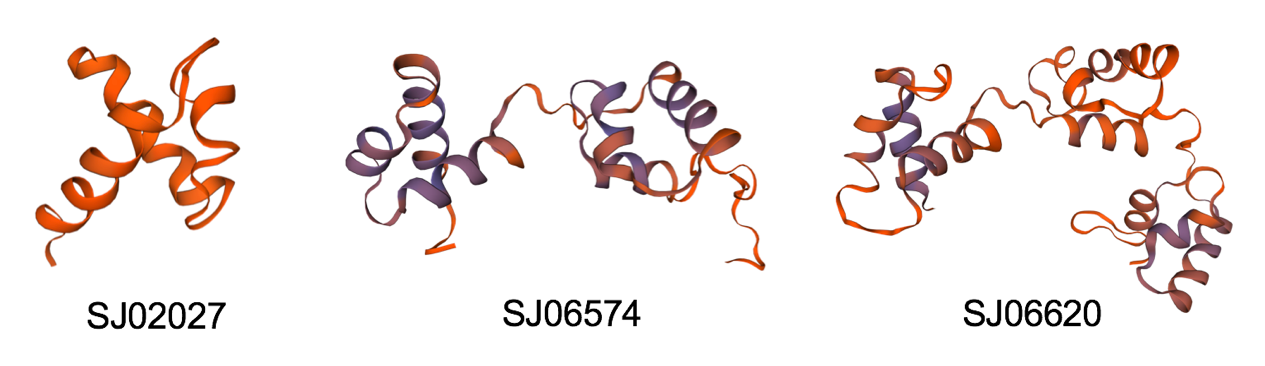


**Supplementary figure S6.** Modeled 3D structures of the MYB domains with one, two or three repeats


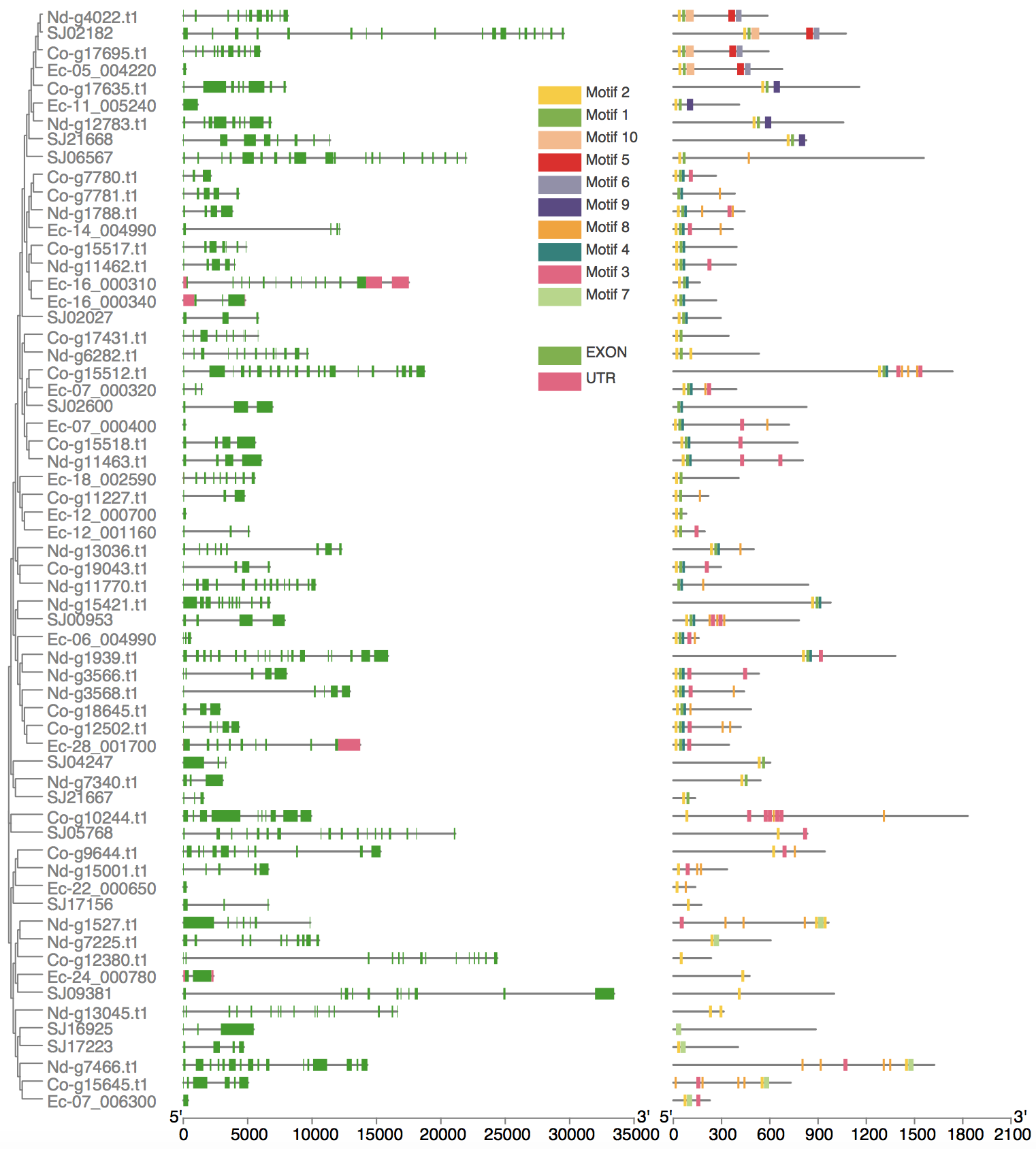


**Supplementary figure S7.** 1R-MYB phylogeny (left), exon/intron structure (middle) and motif composition (right).

**~~
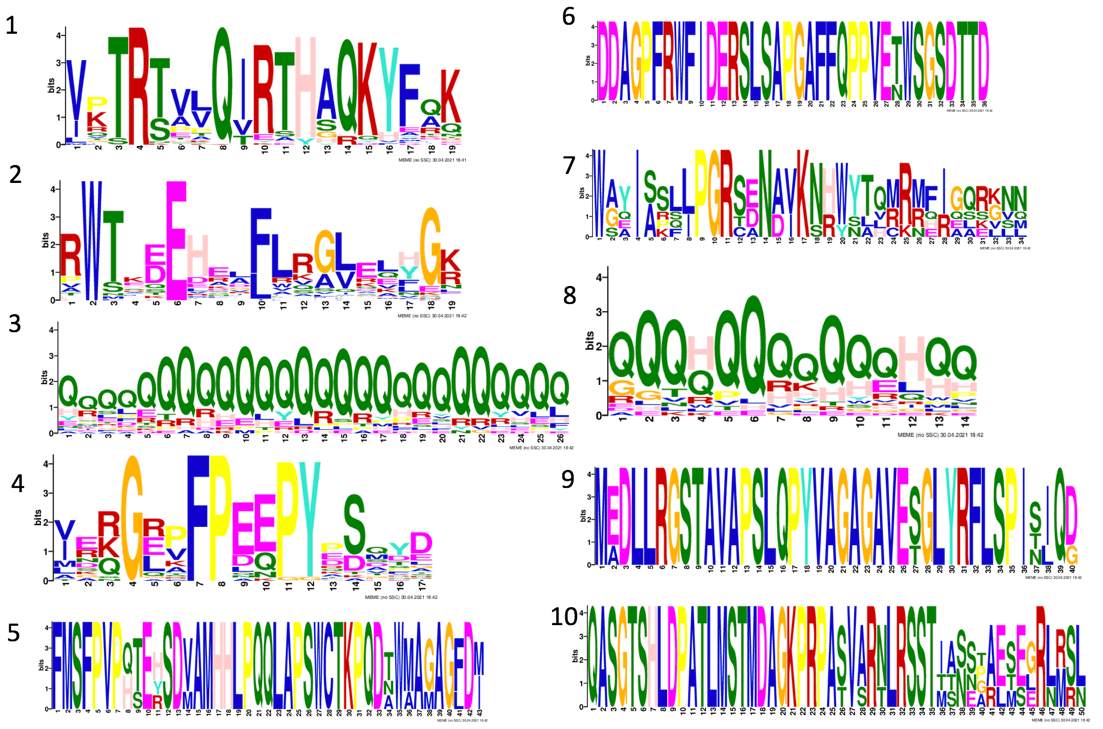
~~**

**Supplementary figure S8.** The ten motifs of 1R-MYBs.


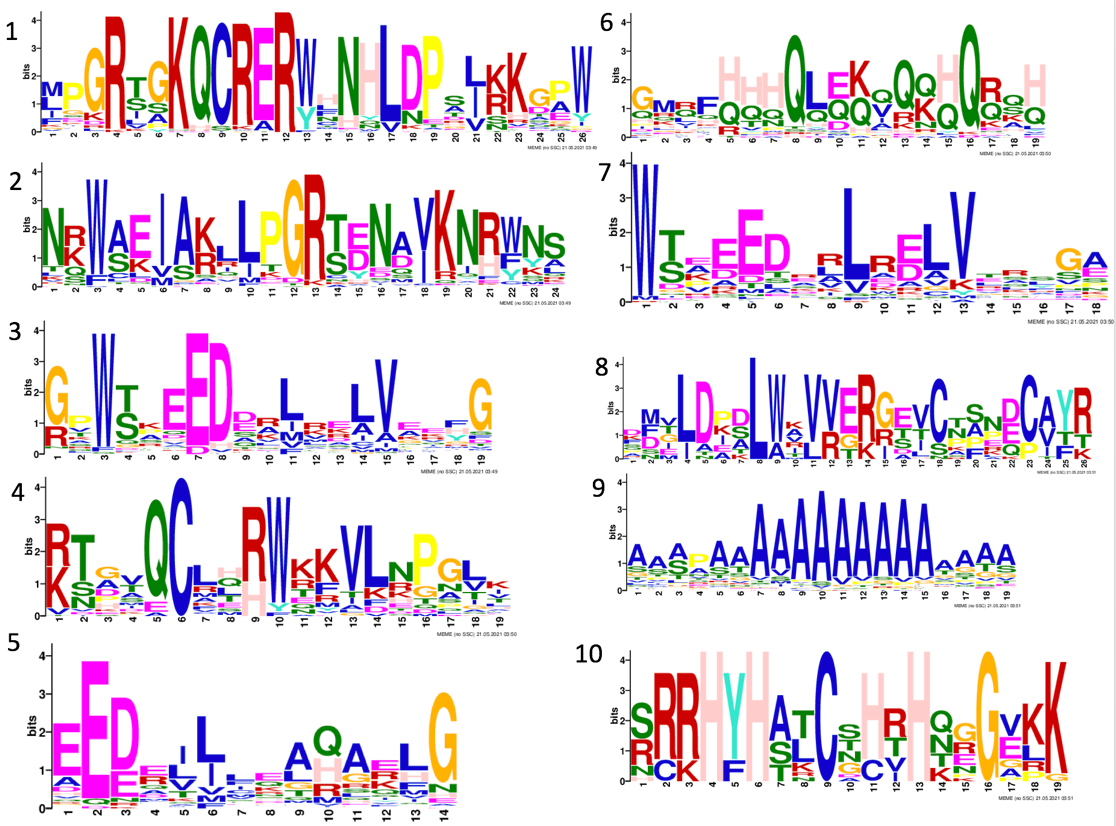


**Supplementary figure S9**. The ten motifs of 2R- and 3R-MYBs.


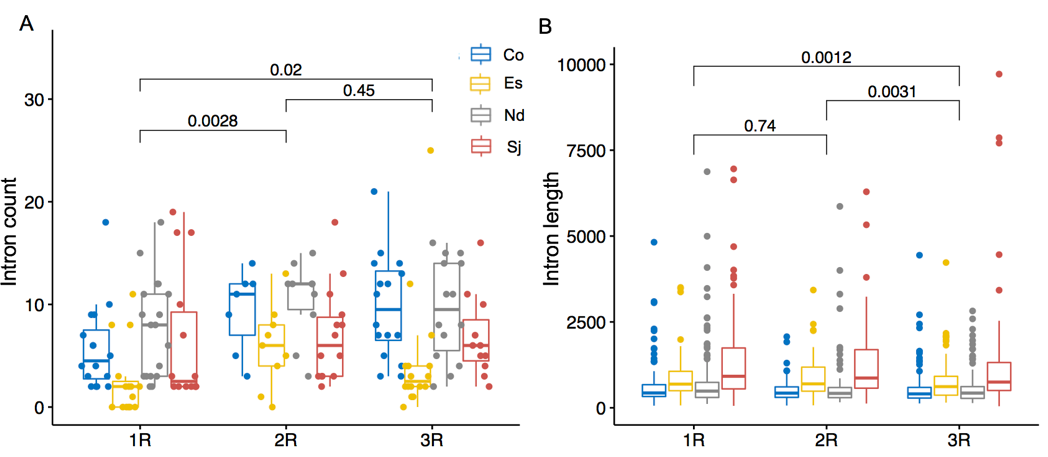


**Supplementary figure S10.** Intron count and length distribution of MYB genes in each brown algal species. The p-value on the plot was calculated using Wilcoxon test.


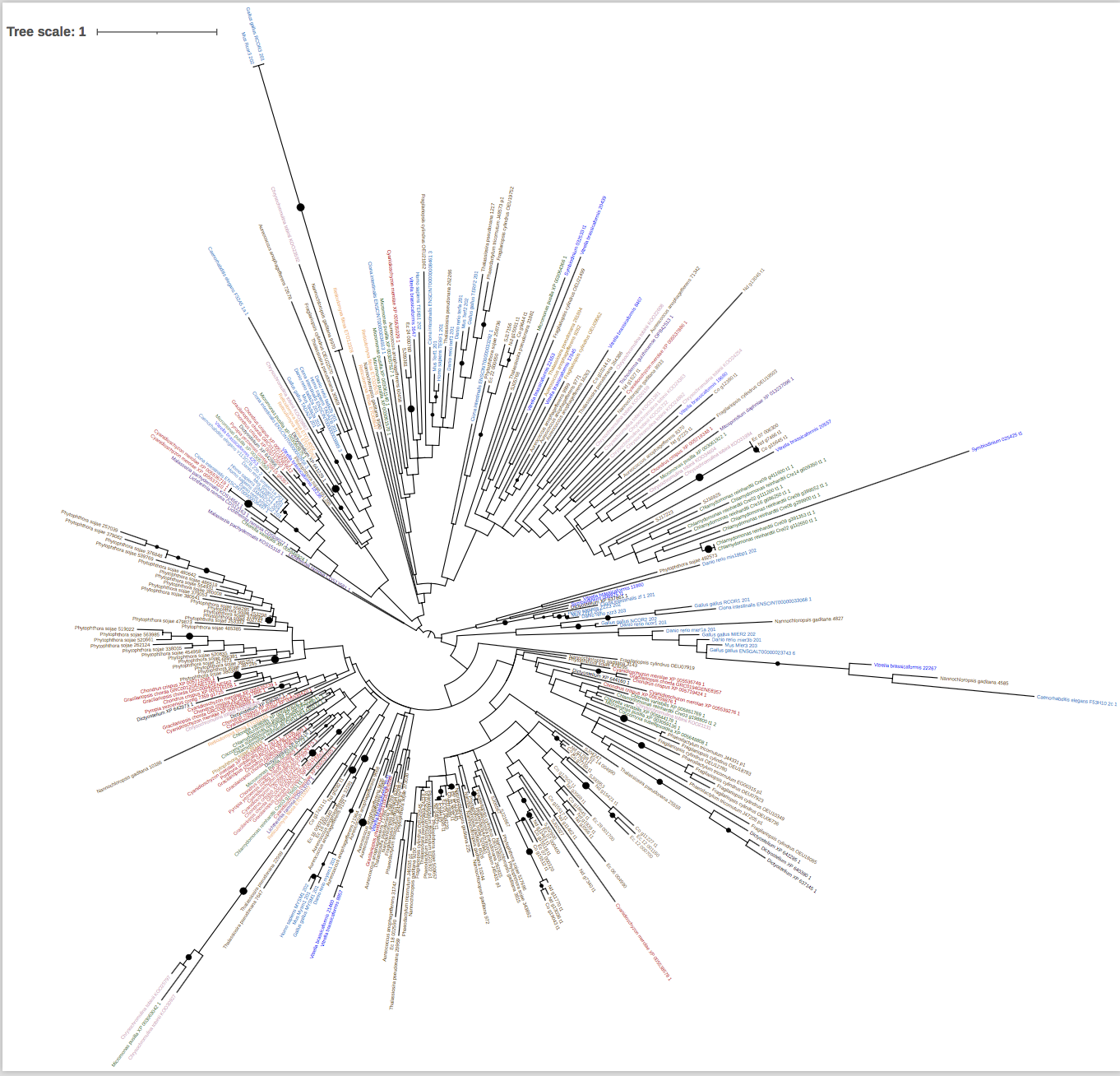


**Supplementary figure S11.** Phylogenetic tree of 1R-MYBs. The tree was constructed based on the alignment of 1R domains, using 52 amino acid sites from 342 sequences. Bootstrap values higher than 50% are displayed as black cycle. The different color labels represent different lineages.


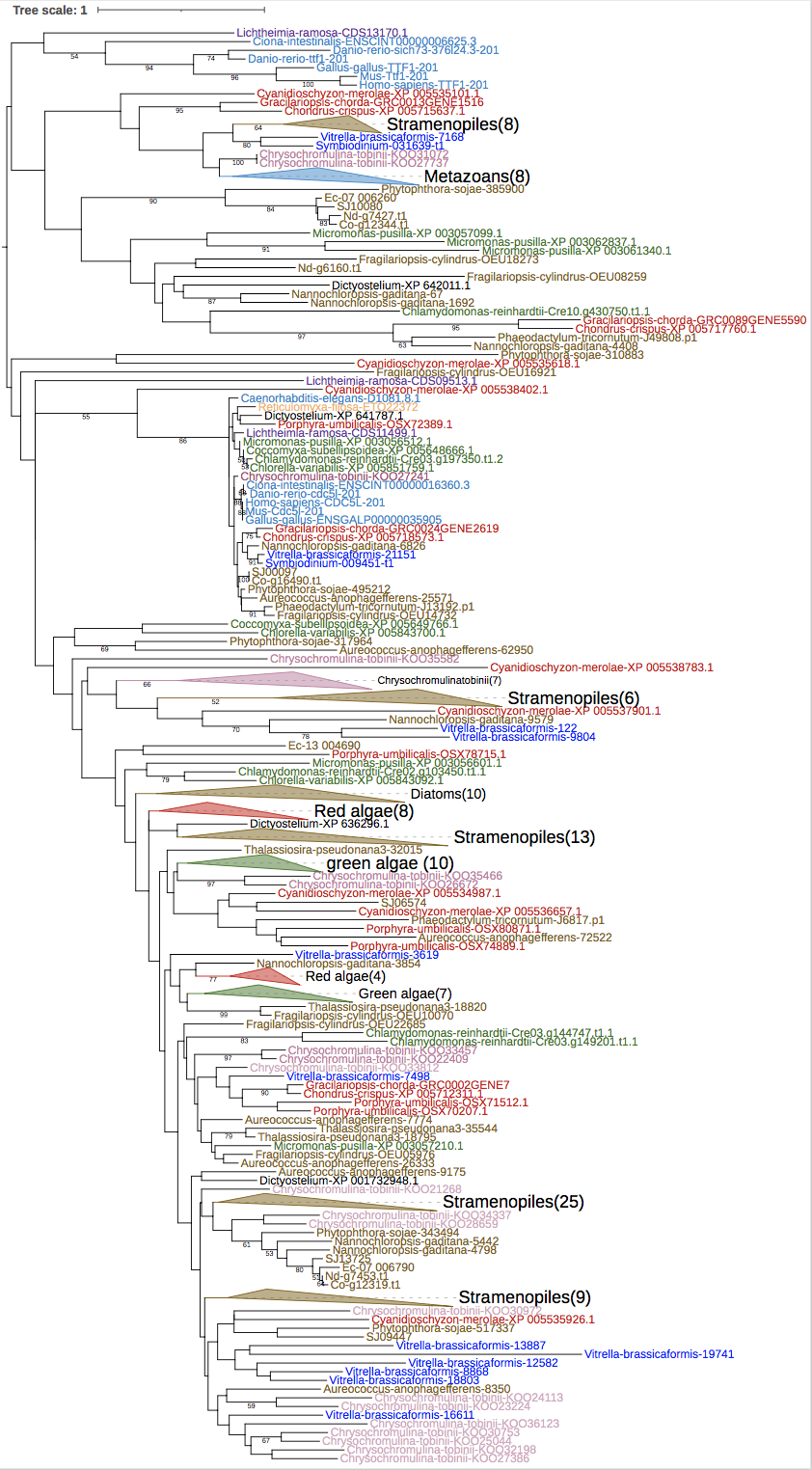


**Supplementary figure S12.** Phylogenetic tree of 2R-MYBs. The tree was constructed based on the alignment of R2R3 domain, using 100 amino acid sites from 256 sequences. Bootstrap values higher than 50% are displayed. The different color labels represent different lineages. Taxonomically homogenous clades are collapsed for better readability of the tree. The numbers in brackets correspond to the number of sequences in the collapsed clades.


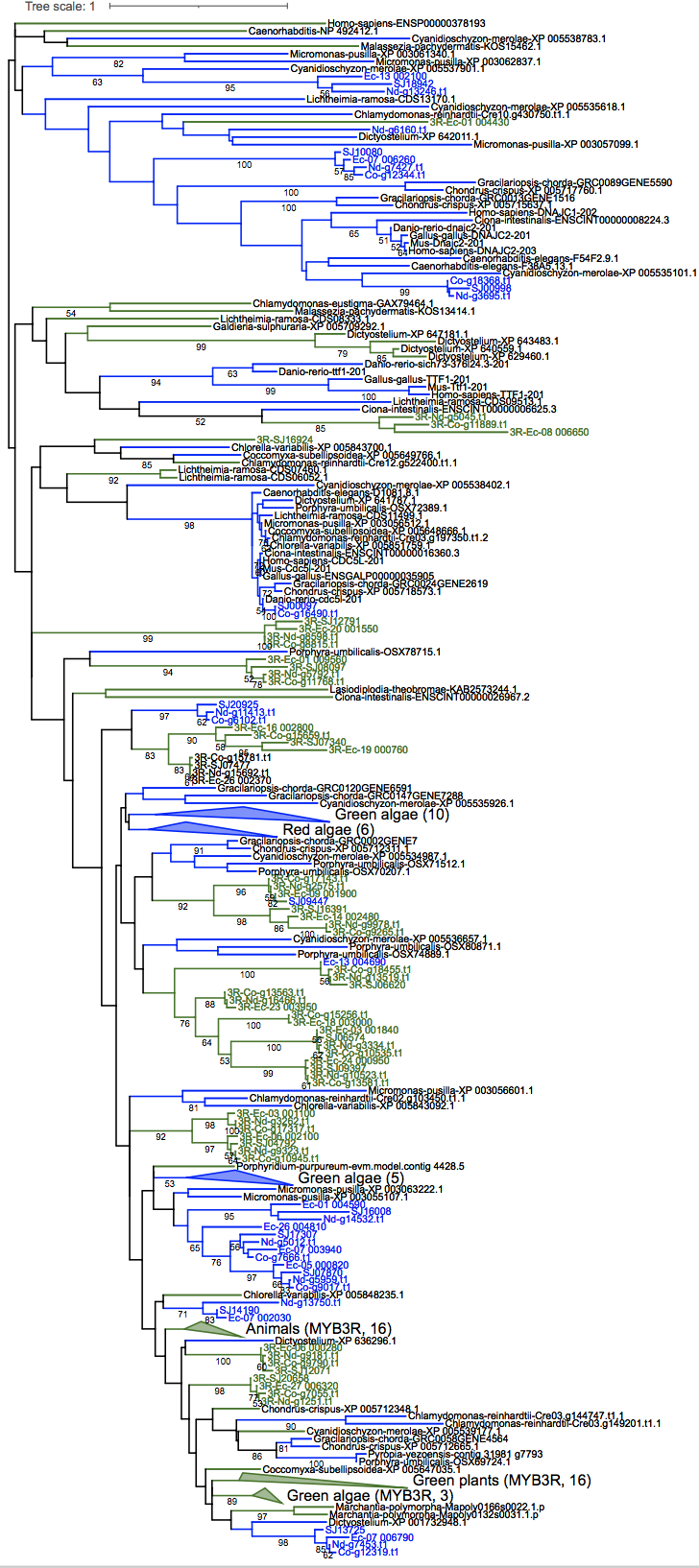


**Supplementary figure S13.** Phylogenetic tree of 2R- and 3R-MYBs. The tree was constructed based on the alignment of R2R3 domains, using 91 amino acid sites from 245 sequences. Bootstrap values higher than 50% are displayed. Taxonomically homogenous clades are collapsed for better readability of the tree. The numbers in brackets correspond to the number of sequences in the collapsed clades. Blue and green branches correspond to 2R-MYBs and 3R-MYBs, respectively. Brown algal 2R and 3R MYB gene IDs are highlighted using blue and green color, respectively.

Supplementary Table **S1**. Sequences information for brown algal MYBs.

*Saccharina japonica*

| Gene ID | Length(AA) | MW(Da) |  | PI |  | Subcellular |
| --- | --- | --- | --- | --- | --- | --- |
|  |  |  | TH |  | SP | localization |
| SJ00097 | 918 | 100012.58 | 0 | 6.77 | N | Nu |
| SJ00953 | 781 | 83263.58 | 0 | 6.45 | N | Nu |
| SJ00998 | 481 | 54308.66 | 0 | 8.84 | N | Cp、Nu |
| SJ02027 | 295 | 30845.72 | 0 | 5.40 | N | Nu |
| SJ02182 | 1073 | 112945.26 | 0 | 5.84 | Y | Nu |
| SJ02600 | 828 | 82630.45 | 0 | 5.05 | N | Nu |
| SJ04247 | 603 | 61626.35 | 0 | 9.07 | N | Nu |
| SJ04792 | 640 | 63901.18 | 0 | 5.05 | N | Nu |
| SJ05768 | 833 | 85794.17 | 0 | 6.94 | N | Nu |
| SJ06567 | 1557 | 146729.52 | 0 | 5.91 | N | Nu |
| SJ06574 | 426 | 41559.95 | 0 | 6.17 | N | Nu |
| SJ06620 | 1059 | 109838.14 | 0 | 8.28 | N | Nu |
| SJ07340 | 360 | 38674.92 | 0 | 10.08 | N | Nu |
| SJ07453 | 978 | 106882.93 | 0 | 6.82 | N | Nu |
| SJ07477 | 1073 | 112356.66 | 0 | 9.47 | N | Nu |
| SJ07870 | 442 | 45194.32 | 0 | 10.56 | N | Nu |
| SJ08097 | 366 | 38839.44 | 0 | 10.80 | N | Cp、Nu |
| SJ09397 | 904 | 96820.92 | 0 | 8.24 | N | Cp、Nu |
| SJ09381 | 999 | 110175.88 | 0 | 6.60 | N | Nu |
| SJ09447 | 891 | 87588.97 | 0 | 9.09 | N | Nu |
| SJ10080 | 619 | 63236.84 | 0 | 9.95 | N | Nu |
| SJ12071 | 144 | 16568.66 | 0 | 6.43 | N | Nu |
| SJ12791 | 683 | 67647.63 | 0 | 10.64 | N | Nu |
| SJ13725 | 978 | 95963.15 | 0 | 5.77 | N | Cw、Cp、Nu |
| SJ14190 | 635 | 61715.22 | 0 | 5.83 | N | Nu |
| SJ16008 | 896 | 90020.78 | 0 | 5.63 | N | Nu |
| SJ16391 | 720 | 74768.34 | 0 | 8.91 | N | Cp、Nu |
| SJ16924 | 433 | 45249.26 | 0 | 7.95 | N | Nu |
| SJ16925 | 885 | 84655.57 | 0 | 4.13 | N | Nu |
| SJ17156 | 175 | 18669.83 | 0 | 4.84 | N | Nu |
| SJ17223 | 402 | 38206.13 | 0 | 10.92 | N | Nu |
| SJ17307 | 1031 | 104645.49 | 0 | 10.17 | N | Nu |
| SJ18942 | 719 | 77262.90 | 0 | 5.13 | N | Nu |
| SJ20658 | 513 | 55431.60 | 0 | 6.18 | N | Nu |
| SJ20925 | 484 | 51288.78 | 0 | 7.34 | N | Cm、Cp、Nu |
| SJ21667 | 136 | 14989.03 | 0 | 11.11 | N | Nu |
| SJ21668 | 826 | 77486.43 | 0 | 11.82 | N | Nu |

*Cladosiphon okamuranus*

| Gene ID | Length(AA) | MW(Da) | TH | PI | SP | Subcellular localization |
| --- | --- | --- | --- | --- | --- | --- |
| g6102.t1 | 618 | 66667.05 | 0 | 5.58 | - | Nu |
| g7055.t1 | 1008 | 105060.89 | 0 | 9.01 | - | Cp、Nu |
| g7666.t1 | 1241 | 130244.87 | 0 | 8.87 | - | Nu |
| g7780.t1 | 265 | 28671.70 | 0 | 8.89 | - | Nu |
| g7781.t1 | 382 | 40111.53 | 0 | 5.55 | - | Nu |
| g8092.t3 | 813 | 88173.75 | 0 | 6.91 | - | Cp、Nu |
| g8475.t1 | 1130 | 120839.38 | 0 | 9.47 | - | Nu |
| g8815.t1 | 1848 | 185463.41 | 0 | 8.80 | - | Nu |
| g9017.t1 | 1690 | 170801.14 | 0 | 9.72 | - | Nu |
| g9265.t1 | 628 | 65436.94 | 0 | 6.64 | - | Cp、Nu |
| g9305.t1 | 1534 | 159688.13 | 0 | 5.30 | - | Nu |
| g9644.t1 | 942 | 98565.38 | 9 | 5.35 | - | Nu |
| g9790.t1 | 1187 | 122432.93 | 0 | 9.53 | - | Nu |
| g10244.t1 | 1831 | 189690.58 | 6 | 8.33 | - | Nu |
| g10535.t1 | 1134 | 115130.31 | 0 | 9.05 | - | Nu |
| g10945.t | 671 | 68847.37 | 0 | 6.60 | - | Nu |
| g11227.t1 | 218 | 23401.34 | 0 | 4.58 | - | Nu |
| g11768.t1 | 763 | 78675.21 | 0 | 6.15 | - | Cp、Nu |
| g11889.t1 | 663 | 73676.13 | 0 | 9.65 | - | Cp、Nu |
| g12319.t1 | 819 | 82880.21 | 0 | 9.35 | - | Nu |
| g12344.t1 | 877 | 91177.12 | 0 | 10.07 | - | - |
| g12380.t1 | 235 | 25788.78 | 0 | 8.82 | - | Nu |
| g12502.t1 | 419 | 43614.12 | 0 | 6.70 | - | Nu |
| g13384.t1 | 786 | 85774.45 | 0 | 6.26 | - | Cp、Nu |
| g13563.t1 | 1687 | 170376.15 | 0 | 10.31 | - | Nu |
| g13581.t1 | 846 | 90221.61 | 0 | 8.82 | - | Nu |
| g15256.t1 | 1509 | 157551.35 | 0 | 9.16 | - | Nu |
| g15512.t1 | 1736 | 179439.69 | 0 | 7.79 | - | Nu |
| g15517.t1 | 393 | 41219.95 | 0 | 6.02 | - | Nu |
| g15518.t1 | 773 | 77325.17 | 0 | 5.70 | - | Nu |
| g15645.t1 | 730 | 77404.44 | 0 | 9.49 | - | Nu |
| g15659.t1 | 1344 | 137351.85 | 0 | 9.93 | - | Nu |
| g15781.t1 | 876 | 92853.64 | 0 | 8.73 | - | Nu |
| g16199.t1 | 741 | 84858.79 | 0 | 5.18 | - | Nu |
| g16490.t1 | 436 | 48335.96 | 0 | 9.54 | - | Nu |
| g17143.t1 | 724 | 75721.14 | 0 | 9.42 | - | Cp、Nu |
| g17317.t1 | 1355 | 134699.35 | 0 | 8.67 | - | Nu |
| g17431.t1 | 344 | 36696.53 | 0 | 9.36 | - | Nu |
| g17635.t1 | 1157 | 107798.70 | 0 | 5.85 | - | Nu |
| g17695.t1 | 592 | 61587.42 | 0 | 5.01 | - | Nu |
| g18368.t1 | 648 | 72645.75 | 0 | 5.68 | - | Cp、Er、Nu |
| g18455.t1 | 1685 | 172479.13 | 0 | 9.02 | - | Nu |
| g18645.t1 | 483 | 49632.79 | 0 | 9.15 | - | Nu |
| g19043.t1 | 296 | 31037.72 | 0 | 5.06 | - | Nu |

*Ectocarpus siliculosus*

| Gene ID | Length(AA) | MW(Da) | TH | PI | SP | MYB-domain | Subcellular localization |  |
| --- | --- | --- | --- | --- | --- | --- | --- | --- |
|  |  |  |  |  |  |  |  |  |
| Ec-01_004430 | 433 | 46876.01 | 0 | 9.83 | — | 3R | Nu |  |
| Ec-01_004590 | 395 | 40832.95 | 0 | 5.31 | — | 2R | Nu、Cp |  |
| Ec-01_009560 | 753 | 78935.22 | 0 | 9.09 | — | 3R | Nu、Cp |  |
| Ec-03_001100 | 1483 | 150015.9 | 0 | 6.18 | — | 3R | Nu |  |
| Ec-03_001260 | 768 | 84522.88 | 0 | 8.65 | — | 4R | Nu、Cp |  |
| Ec-03_001840 | 930 | 95398.61 | 0 | 9.72 | — | 3R | Nu、Cp |  |
| Ec-04_003360 | 939 | 95873.88 | 0 | 5.14 | — | 5R | Nu |  |
| Ec-05_000820 | 1862 | 187310.4 | 0 | 9.86 | — | 2R | Nu |  |
| Ec-05_004220 | 676 | 70069.17 | 0 | 5.26 | — | 1R | Nu |  |
| Ec-06_000280 | 1147 | 117502.6 | 0 | 9.65 | — | 3R | Nu |  |
| Ec-06_002100 | 522 | 55290.65 | 0 | 10.65 | — | 3R | Nu |  |
| Ec-06_004990 | 157 | 18596.47 | 0 | 6.23 | — | 1R | Nu |  |
| Ec-06_008700 | 501 | 55716.9 | 0 | 6.35 | — | 3R | Nu |  |
| Ec-07_000320 | 391 | 42770.83 | 0 | 5.14 | — | 1R | Cp |  |
| Ec-07_000400 | 718 | 73534.65 | 0 | 5.21 | — | 1R | Nu |  |
| Ec-07_002030 | 429 | 46278.5 | 0 | 10.81 | — | 2R | Nu、Cp |  |
| Ec-07_003940 | 444 | 47878.71 | 0 | 9.67 | — | 2R | Nu |  |
| Ec-07_006260 | 401 | 41631.36 | 0 | 10.52 | — | 2R | Nu |  |
| Ec-07_006300 | 226 | 24420.95 | 0 | 9.84 | — | 1R | Nu |  |
| Ec-07_006790 | 663 | 67863.53 | 0 | 9.98 | — | 2R | Nu、Cp |  |
| Ec-08_006650 | 512 | 57875.36 | 0 | 10.43 | — | 1R | Nu |  |
| Ec-09_001900 | 778 | 78036.07 | 0 | 9.91 | — | 3R | Nu |  |
| Ec-11_005240 | 408 | 40837.63 | 0 | 6.08 | — | 1R | Nu |  |
| Ec-12_000700 | 79 | 8738.62 | 0 | 4.43 | — | 1R | Nu、Cp |  |
| Ec-12_001160 | 194 | 21227.31 | 0 | 5.53 | — | 1R | Nu |  |
| Ec-13_002100 | 703 | 74478.85 | 0 | 5.07 | — | 2R | Nu |  |
| Ec-13_004690 | 1188 | 121737.5 | 0 | 7.43 | — | 3R | Nu |  |
| Ec-14_002480 | 303 | 34283.91 | 2 | 9.91 | — | 3R | Nu、Cp |  |
| Ec-14_004990 | 369 | 38714.8 | 0 | 5.27 | — | 1R | Nu |  |
| Ec-16_000310 | 164 | 17283.42 | 0 | 6.05 | — | 1R | Nu |  |
| Ec-16_000340 | 265 | 27868.65 | 0 | 4.98 | — | 1R | Nu |  |
| Ec-16_002800 | 1891 | 196980.4 | 0 | 8.52 | — | 4R | Nu |  |
| Ec-18_002590 | 405 | 44786.66 | 0 | 4.9 | — | 2R | Nu |  |
| Ec-18_003000 | 470 | 51363.56 | 0 | 9.91 | — | 3R | Nu |  |
| Ec-19_000760 | 398 | 44470.68 | 0 | 10.84 | — | 3R | Nu、Cp |  |
| Ec-20_001550 | 604 | 63560.73 | 0 | 9.64 | — | 3R | Nu |  |
| Ec-22_000650 | 136 | 14211.42 | 0 | 6.28 | — | 1R | Nu |  |
| Ec-22_002950 | 852 | 92036.1 | 0 | 9.14 | — | 6R | Nu、Cp |  |
| Ec-23_003950 | 1748 | 176088.5 | 0 | 10.59 | — | 3R | Nu |  |
| Ec-24_000780 | 473 | 52335.36 | 0 | 8.89 | — | 1R | Nu |  |
| Ec-24_000950 | 668 | 72192.51 | 0 | 9.15 | — | 3R | Cp |  |
| Ec-26_002370 | 923 | 97618.83 | 0 | 8.24 | — | 3R | Nu |  |
| Ec-26_004810 | 151 | 17397.59 | 0 | 9.32 | — | 2R | Nu |  |
| Ec-27_006320 | 933 | 95408.64 | 0 | 9.75 | — | 3R | Nu |  |
| Ec-28_001700 | 345 | 36543.3 | 0 | 5.45 | — | 1R | Nu、Cm |  |

Note: Cp: cytoplasmic Nu: nuclear

*Nemacystus decipiens*

| Gene ID | Length(AA) | MW(Da) | TH | PI | MYB-domain | SP | Subcellular localization |
| --- | --- | --- | --- | --- | --- | --- | --- |
| g1251.t1 | 921 | 96083.27 | 0 | 8.85 | 3R | － | Cp、Ec、Nu |
| g1527.t1 | 965 | 99690.69 | 0 | 9.03 | 1R | － | Nu |
| g1788.t1 | 443 | 47250.09 | 0 | 6.17 | 1R | － | Nu |
| g1939.t1 | 1381 | 147035.37 | 0 | 9.42 | 2R | － | Cp、Nu |
| g2575.t1 | 770 | 79221.89 | 0 | 9.67 | 3R | － | Nu |
| g3262.t1 | 1270 | 130980.77 | 6 | 5.42 | 3R | － | Ec、Nu |
| g3266.t1 | 795 | 87400.16 | 0 | 8.95 | 5R | － | Nu |
| g3334.t1 | 1076 | 109313.73 | 0 | 9.72 | 3R | － | Nu |
| g3566.t1 | 532 | 56817.81 | 0 | 5.14 | 1R | － | Ec、Nu |
| g3568.t1 | 440 | 46363.90 | 0 | 6.14 | 1R | － | Ec、Nu |
| g3695.t1 | 638 | 71931.08 | 0 | 5.82 | 3R | － | Cp、Er、Nu |
| g4022.t1 | 586 | 60958.71 | 0 | 4.97 | 1R | － | Ec、Nu |
| g4149.t1 | 1662 | 174223.53 | 0 | 5.43 | 6R | － | Ec |
| g5012.t1 | 1257 | 130874.35 | 0 | 8.78 | 2R | － | Nu |
| g5045.t1 | 561 | 62689.80 | 0 | 9.29 | 3R | － | Nu |
| g5792.t1 | 710 | 73277.49 | 0 | 6.24 | 3R | － | Cp、Nu |
| g5959.t1 | 1769 | 179503.60 | 0 | 9.46 | 2R | － | Ec、Nu |
| g6160.t1 | 767 | 83802.00 | 0 | 5.67 | 2R | － | Cp |
| g6282.t1 | 532 | 56223.22 | 0 | 9.46 | 1R | － | Nu |
| g7225.t1 | 605 | 62041.34 | 0 | 4.98 | 1R | － | Nu |
| g7340.t1 | 542 | 58138.08 | 0 | 5.54 | 1R | － | Nu |
| g7343.t1 | 677 | 73304.86 | 0 | 8.99 | 1R | － | Nu |
| g7427.t1 | 931 | 95022.60 | 0 | 9.86 | 2R | － | Nu |
| g7453.t1 | 814 | 81817.53 | 0 | 7.35 | 2R | － | Nu |
| g7466.t1 | 1623 | 171532.49 | 1 | 8.90 | 1R | － | Nu |
| g9181.t1 | 1257 | 128993.22 | 0 | 9.63 | 3R | － | Nu |
| g9230.t1 | 942 | 100889.42 | 0 | 9.2 | 6R | － | Cp、Nu |
| g9323.t1 | 663 | 68592.00 | 0 | 5.79 | 3R | － | Cp、Ec、Nu |
| g9978.t1 | 641 | 66789.60 | 0 | 8.03 | 3R | － | Nu |
| g10523.t1 | 834 | 88747.94 | 0 | 8.99 | 3R | － | Cp、Nu |
| g11413.t1 | 447 | 48304.38 | 0 | 4.98 | 2R | － | Nu |
| g11462.t1 | 389 | 40534.90 | 0 | 5.63 | 1R | － | Nu |
| g11463.t1 | 805 | 79992.46 | 0 | 6.45 | 1R | － | Ec、Nu |
| g11770.t1 | 839 | 91482.97 | 0 | 7.91 | 1R | － | Chl、Ec、Mt |
| g12783.t1 | 1057 | 100166.92 | 0 | 6.05 | 1R | － | Nu |
| g13036.t1 | 500 | 52813.35 | 0 | 5.73 | 1R | － | Cm、Nu |
| g13045.t1 | 313 | 34718.15 | 0 | 5.32 | 2R | － | Cp、Nu |
| g13246.t1 | 490 | 54177.05 | 0 | 5.32 | 2R | － | Nu |
| g13519.t1 | 1256 | 126545.51 | 0 | 5.95 | 3R | － | Ec、Nu |
| g13750.t1 | 745 | 80568.43 | 0 | 5.43 | 3R | － | Cp、Nu |
| g14517.t1 | 1243 | 132252.43 | 0 | 8.68 | 6R | － | Nu |
| g14532.t1 | 1967 | 205145.87 | 9 | 6.68 | 2R | － | Cp、Nu |
| g15001.t1 | 334 | 34981.27 | 0 | 5.82 | 1R | － | Nu |
| g15421.t1 | 978 | 106933.35 | 0 | 5.92 | 1R | － | Nu |
| g15692.t1 | 738 | 78625.67 | 0 | 6.58 | 3R | － | Cp、Nu |
| g16466.t1 | 1023 | 105201.20 | 0 | 10.21 | 3R | － | Nu |
